# Perioperative patient safety recommendations: systematic review of clinical practice guidelines

**DOI:** 10.1093/bjsopen/zrae143

**Published:** 2024-12-11

**Authors:** Ismael Martinez-Nicolas, Daniel Arnal-Velasco, Eva Romero-García, Neus Fabregas, Yolanda Sanduende Otero, Irene Leon, Ashish A Bartakke, Javier Silva-Garcia, Anna Rodriguez, Claudia Valli, Sandro Zamarian, Adam Zaludek, Jose Meneses-Echavez, Andrés F Loaiza-Betancur, Paulo Sousa, Carola Orrego, Victor Soria-Aledo, SAFEST Consortium, Joaquim Baneres, Genis Carrasco, Rosa Sunol, Helena Vall, Hiske Calsbeek, Yvette Emond, Anita Heideveld-Chevalking, Pedro Casaca-Carvalho, Andreia Leite, Ana Beatriz Nunes, Ayshe Seyfulayeva, Edoardo De Robertis, Cathy Weynants, Maria Wittman, Pascal Garel, Marie Nabbe, Oliver Groene, Sophie Wang, Mari Kangasniemi, Janne Kommusaar, Kaja Kristensen, Kaja Polluste, Janne Pühvel, Joel Starkopf, David Marx, Frantisek Vlcek, Willemijn Schäfer, Caroline Schlinkert, Nina van der Schoot, Lilian Van Tuyl, Marieke Voshaar, Cordula Wagner

**Affiliations:** Spanish Anaesthesia and Reanimation Incident Reporting System (SENSAR), Alcorcon, Spain; Spanish Anaesthesia and Reanimation Incident Reporting System (SENSAR), Alcorcon, Spain; Department of Anaesthesia and Reanimation, Hospital Universitario Fundación Alcorcon, Alcorcon, Spain; Spanish Anaesthesia and Reanimation Incident Reporting System (SENSAR), Alcorcon, Spain; Department of Anaesthesia and Reanimation, Hospital Univesitari Policlinic La Fe, Valencia, Spain; Spanish Anaesthesia and Reanimation Incident Reporting System (SENSAR), Alcorcon, Spain; Department of Anaesthesia and Reanimation, Hospital Clínic, Barcelona, Spain; Spanish Anaesthesia and Reanimation Incident Reporting System (SENSAR), Alcorcon, Spain; Department of Anaesthesia and Reanimation, Hospital Universitario de Pontevedra, Pontevedra, Spain; Spanish Anaesthesia and Reanimation Incident Reporting System (SENSAR), Alcorcon, Spain; Department of Anaesthesia and Reanimation, Hospital Clínic, Barcelona, Spain; Spanish Anaesthesia and Reanimation Incident Reporting System (SENSAR), Alcorcon, Spain; Department of Anaesthesia and Reanimation, Hospital Valle de Los Pedroches, Pozoblanco, Spain; Spanish Anaesthesia and Reanimation Incident Reporting System (SENSAR), Alcorcon, Spain; Department of Anaesthesia and Reanimation, Hospital Universitario 12 de Octubre, Madrid, Spain; Avedis Donabedian Research Institute, Barcelona, Spain; Universidad Autónoma de Barcelona, Spain; Avedis Donabedian Research Institute, Barcelona, Spain; Universidad Autónoma de Barcelona, Spain; Spojená Akreditační Komise, Prague, Czech Republic; Spojená Akreditační Komise, Prague, Czech Republic; Third Faculty of Medicine, Department of Public Health, Charles University, Prague, Czech Republic; Norwegian Institute of Public Health, Oslo, Norway; Instituto Universitario de Educación Física, Universidad de Antioquia, Medellín, Colombia; NOVA National School of Public Health, Comprehensive Health Research Center, CHRC, NOVA University, Lisbon, Portugal; Avedis Donabedian Research Institute, Barcelona, Spain; Universidad Autónoma de Barcelona, Spain; Network for Research on Chronicity, Primary Care, and Health Promotion (RICAPPS), Barcelona, Spain; Spanish Anaesthesia and Reanimation Incident Reporting System (SENSAR), Alcorcon, Spain; Department of Surgery, Hospital Universitario Morales Meseguer, Murcia, Spain

## Abstract

**Background:**

Surgical-related incidents are a common cause of in-hospital adverse events. Surgical patient safety would benefit from evidence-based practices, but a comprehensive collection of patient safety recommendations is still lacking. This study aimed to compile and assess the perioperative patient safety recommendations for adults.

**Method:**

A systematic review of clinical practice guidelines was conducted using Medline, Embase, Cochrane, Virtual Health Library Regional Portal, and Trip Database from 2012 to 2022. Eligibility criteria followed a PICAR strategy for patient safety recommendations in the perioperative care continuum. Guidelines were appraised for quality, particularly focusing on the ‘rigour of development’ domain of the AGREE-II tool for those containing strong recommendations. Descriptive analyses were conducted, emphasizing guideline quality, recommendation strength, and the supporting level of evidence.

**Results:**

From the 267 guidelines, 4666 perioperative patient safety recommendations were extracted, of which 44.9% (2095) were strongly recommended. Of these, 322 had the highest level of evidence, but only 18 guidelines met high standards in the AGREE-II ‘rigour of development’ domain. A subset of 78 recommendations ranked the highest in the strength of recommendation, level of evidence, and rigour of development of their guidelines. A gap was found within pre-admission and post-discharge care recommendations.

**Discussion:**

This review highlights the noteworthy variability in the methodological quality of the guidelines, and a discordance between strength of recommendation and evidence level of the available perioperative patient safety recommendations. These findings provide valuable information for advising policy decisions and promoting best practices to enhance global surgical safety.

**Registration:**

PROSPERO (CRD42022347449).

## Teaser

This systematic review of clinical practice guidelines extracted 4666 patient safety recommendations for perioperative care from 267 studies. A subset of recommendations ranked high in strength, level of evidence, and rigour of development. These findings are valuable for policy decisions and promoting best practices to enhance global surgical care safety.

## Introduction

Surgical-related safety incidents are among the most prevalent in-hospital adverse events, accounting for a median of 40% of the total (range 27–75%)^1^. Preventable harm is more prevalent in patients treated in intensive care and surgical units^2^. Although an astonishing global decrease in perioperative mortality rate has occurred over the last 50 years, a push to implement evidence-based best practice is still needed^3^, as well as evidence-based directions for designing and prioritizing efficient mitigation strategies^2^.

The adoption of evidence-based practices can significantly improve the safety outcomes of surgical care^4,5^. However, translating evidence into practice is slow in the healthcare field^6^, and the available patient safety recommendations are currently scattered among multiple guidelines from international, national, and regional contexts, many of which have been published in the grey literature. Clinicians and managers face the challenge of prioritizing practices to be implemented among multiple publishers and references^7^. A recent evaluation and ranking of patient safety practices related to surgical patients in Europe revealed a divergence in expert views and a lack of robust research evidence for many practices^8^.

The aim of this study was to compile and describe the available patient safety recommendations along the perioperative care continuum for the adult population. A secondary objective was to examine the characteristics and quality of the guidelines and identify a selection of evidence-based and strongly recommended perioperative patient safety practices.

## Methods

A systematic review of clinical practice guidelines was performed following the methodological recommendations published by Johnston *et al*.^9^. This systematic review was registered in the PROSPERO International prospective register of systematic reviews and adhered to the PRISMA 2020 statement (*Supplementary Materials A1*)^10^.

### Eligibility criteria

Following a detailed PICAR (Population, Intervention, Comparators, Attributes of eligibility, and Recommendation characteristics) strategy (*Supplementary Materials M1*)^9^, a comprehensive database search that included any clinical practice guidelines (CPG), position statement, expert consensus, or other publication (hereafter described as ‘guidelines’), published from 2012 to 2022, containing at least one surgical patient safety recommendation in the perioperative continuum of care for adult population (>18 years) was conducted.

Exclusion criteria were as follows: documents that did not properly contain recommendations; previous versions of an already included guideline; non-original guidelines (that is translations or adaptations of other currently available guidelines); guidelines developed for a single healthcare institution rather than a multi-institutional (international, national, or regional) scope; technical guidelines on surgical or anaesthetic procedures; and guidelines about percutaneous procedures and minor surgeries, procedures performed before the surgical indication (that is screening and diagnosis procedures) or related to uncommon situations or low prevalent procedures out of a broader scope (for example guidelines in a COVID-19 context, or robotic pancreatoduodenectomy). No language, geographical origin, organization, or guideline-type restrictions were applied.

A CPG was defined as a systematically developed document created with a validated methodology, which includes identifying the literature on specific clinical question(s), characterized by explicit methods of searching, selecting, and grading the available evidence; position statement as a document that elucidates, justifies, and recommends a particular approach to a clinical problem, typically outlining the organization's or group’s stance on the matter; and expert consensus as a document with recommendations developed based on a collective opinion or consensus of the convened expert panel^11^.

Surgical patient safety recommendations were defined as those that aim to prevent harm or reduce the incidence of preventable morbidity and mortality and may be classified into one of 15 predefined surgical safety areas (*Supplementary Materials M2*). Finally, the perioperative continuum was defined as five periods of time, from the surgical indication until 90 days after surgery: Pre-admission; Preoperative; Intraoperative; Postoperative; and Post-discharge.

### Information sources and search strategy

A structured bibliographic search was developed by two experienced health librarians, including Thesaurus, and search terms related to perioperative periods, patient safety, and guidelines (*Supplementary Materials M3*). The following databases were used: Medline, Embase, Cochrane, LILACS, IBECS, BDENF, BINACIS, BIGG, SES-SP, and WHO IRIS through the Virtual Health Library (VHL) Regional Portal, and Trip Database.

In addition, an exhaustive search of grey literature was carried out among relevant websites from recognized health and patient safety organizations, regulatory agencies, governmental and non-governmental stakeholders (that is patient safety and patient advisory organizations), and scientific societies. Additionally, in the framework of the SAFEST project^12^, international experts were contacted and invited to provide regional or national perioperative safety-related guidelines.

### Screening

Three independent reviewers (IMN, AZ, and AR) assessed in pairs the titles and abstracts for eligibility using the Rayyan application website^13^. To improve reliability, a pilot screening was conducted using Cohen’s kappa^14^, which obtained a moderate agreement between raters (0.55, 95% c.i. 0.34 to 0.77). Full-text screening followed a procedure similar to that described by the same reviewers. Disagreements were resolved by a fourth reviewer (DAV).

### Data extraction

Following training and calibration, three reviewers (IMN, AB, and JSM) extracted guideline characteristics from the included publications. Three additional senior reviewers (ERS, NF, and YSD) completed the cross-checking. A standardized extraction database was defined. It included guideline identification, year of publication, number of recommendations, type of document^11^, promoting organization, publishing origin, and income level classified using World Bank 2023 data^15^, scope of application, language of publication, and grading system for level of evidence (LE) and strength of recommendation (SR). Links with additional information or materials were collected.

A similar methodology was applied to build a second standardized extraction database for the recommendations. Only unique, non-repeated recommendations were extracted from the guidelines, gathering the following characteristics: verbatim description, perioperative period, clinical setting, surgical safety area and subarea, and reported LE and SR. A detailed description of these databases is available in *Supplementary Materials M4*.

### Qualitative analysis

Given the variability in grading systems, the diverse LE were converted into a normalized grading system, as suggested by Johnston *et al*. (*Supplementary Materials M5*)^9^. The normalized system provides a proxy LE based on the Grades of Recommendation, Assessment, Development, and Evaluation (GRADE) approach^16^, considering the type of study design and, when possible, GRADE’s indications for upgrading and downgrading the evidence level.

The SRs were also normalized into a binary system. Recommendations with the highest strength in their reported grading system or with a high level of consensus—namely, over 70%—were considered ‘Strong’, whereas the rest of the recommendations were classified as ‘Weak and not reported’.

### Quality assessment

A basic quality appraisal of all references was performed, identifying explicitly stated systematic methods^17^, using three criteria: a method based on evidence (that is explicit structured literature review with terms and databases) is described; a method to formulate recommendations (that is explicit grading system or consensus method) is described; and an external review is explicitly described.

Additionally, to identify trustworthy recommendations with a high agreement or a high impact on patient safety, a further quality assessment was performed using the domain of ‘rigour of development’ of the Appraisal of Guidelines Research and Evaluation Instrument, version II (AGREE-II)^18^ to guidelines containing at least a recommendation categorized as ‘Strong’ in the normalized grading system.

Two trained independent reviewers (JM and AL) performed all assessments. Each item was graded on a 7-point scale from 1 (the guideline does not comply with this item at all) to 7 (the guideline fully complies with it). Any differences in scores equal to or greater than 3 points across evaluators were resolved by consensus. A standardized percentage was then calculated for the domain, and the guidelines were classified into three categories: high quality (>70%), moderate quality (50–70%), and low quality (<50%). This percentage was calculated as follows^18^:


$$\frac{Obtained\;score-Minimum\;possible\;score}{Maximum\;possible\;score-Minimum\;possible\;score}\times 100$$

### Statistical analysis

A descriptive analysis was conducted, and selection was performed for both guidelines and recommendations based on the guideline quality, SR, and LE. Frequency tables and comparisons were tested using χ^2^ and Mann–Whitney U tests for nominal and numerical variables respectively. Statistical significance was set at *P* < 0.05. All statistical analyses were conducted using Stata 14.

## Results

### Descriptive results from the included guidelines

**Fig. 1:**
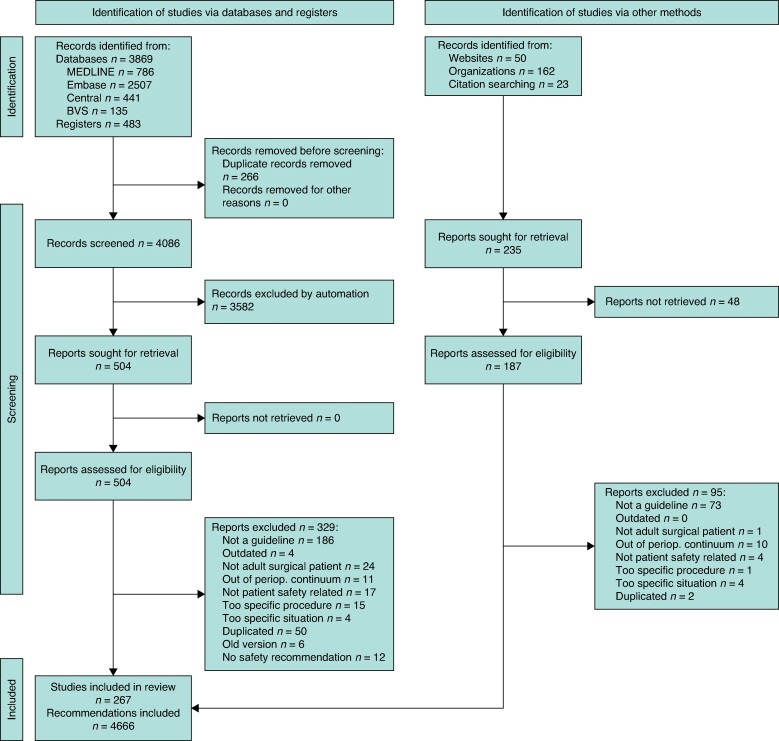
PRISMA 2020 flow diagram for the selection of guidelines

The literature search resulted in 4352 hits from databases and 236 hits from grey literature. A total of 4086 references were screened, and after full-text assessment for eligibility, 267 documents were included in the qualitative synthesis and 4666 perioperative patient safety recommendations were extracted (*Fig. 1*).

*Table 1* shows the main characteristics of the included guidelines. Almost half of all guidelines (47%) were CPG as the type of publication, 195 (73%) were developed from the scientific community, and 197 (74%) were from high-income countries. A reduced group of 14 guidelines^19–32^, potentially applicable to any income level, was developed by supranational organizations. These included the WHO’s ‘Global Guidelines for the Prevention of Surgical Site Infection’^19^, three guidelines from the World Society of Emergency Surgery^20–22^, and four guidelines from the Enhanced Recovery After Surgery (ERAS) Society^23–26^.

**Table 1:** Descriptive characteristics of the guidelines included in the quantitative analysis

	All documents (*n* = 267)	Without any strong PS recommendation (*n* = 158)	With at least one strong PS recommendation (*n* = 109)
Number of recommendations, median (i.q.r.)	20 (10–40)	15 (8–28)	34.5 (19–71.5)
Number of recommendations extracted, median (i.q.r.)	10 (5–17)	9 (4–15)	14 (7–29)
**Type of publication**			
CPG	126 (47.2)	32 (20.3)	94 (86.2)
Expert consensus	52 (19.5)	41 (26.0)	11 (10.1)
Position statements	40 (15)	40 (25.3)	0 (0)
Other	49 (18.4)	45 (28.5)	4 (3.7)
**Type of organization**			
Public institution	41 (15.4)	30 (19.0)	11 (10.1)
Private organization	9 (3.4)	8 (5.1)	1 (0.9)
Scientific community	195 (73)	104 (65.8)	91 (83.5)
Other	22 (8.2)	16 (10.1)	6 (5.5)
**Scope of the guidelines**			
International	82 (30.7)	31 (19.6)	51 (46.8)
National	183 (68.5)	125 (79.1)	58 (53.2)
Subnational	2 (0.8)	2 (1.3)	0 (0)
**Income level of the developer**			
High income	197 (73.8)	134 (84.8)	63 (57.8)
Upper middle income	13 (4.9)	6 (3.8)	7 (6.4)
High and middle income	43 (16.1)	17 (10.8)	26 (23.9)
Any income	14 (5.2)	1 (0.6)	13 (11.9)
**Origin of the developer**			
East Asia and Pacific	15 (5.6)	13 (8.2)	2 (1.8)
Europe and Central Asia	136 (50.9)	87 (55.1)	49 (45)
Latin America and Caribbean	11 (4.1)	4 (2.5)	7 (6.4)
North America	91 (34.1)	53 (33.5)	38 (34.9)
Worldwide organization	14 (5.2)	1 (0.6)	13 (11.9)
**Version of the guidelines**			
New	151 (56.6)	103 (65.2)	48 (44)
Updated	116 (43.5)	55 (34.8)	61 (56)
Structured evidence review	142 (53.2)	50 (31.7)	92 (84.4)
Explicit method for grading recommendations	136 (50.9)	35 (22.2)	101 (92.7)
Externally reviewed	92 (34.5)	29 (18.4)	63 (57.8)

Values are *n* (%) unless indicated otherwise. CPG, clinical practice guideline; PS, patient safety.

The methodology and content of the guidelines were highly heterogeneous. GRADE stood out as the most common method for grading recommendations (in 62, 47% of the guidelines) among the more than 25 different approaches used across guidelines. The number of recommendations per guideline varied substantially (median 20, i.q.r. 10–40) given the diversity of their scope. Regarding the extracted recommendations, the CPG from the Association of PeriOperative Registered Nurses (AORN) contributed the most (450, 10% of all included recommendations)^33^.

Guidelines with at least a strong recommendation were more likely to comply with the three basic quality appraisal criteria (presence of explicit structured evidence review, method for grading recommendations, and external review), and were more frequently catalogued as CPG (Mann–Whitney U and χ^2^ with any *P* < 0.001). The three basic quality appraisal criteria were present simultaneously in 73 (27%) guidelines, of which 55 (75%) had a strong recommendation and 69 (94%) complied with the CPG definition.

### Descriptive results from the extracted recommendations

*Table 2* presents the characteristics of the extracted perioperative patient safety recommendations. Most of the recommendations (1664, 36%) were applicable to more than one phase of the perioperative continuum, whereas the intraoperative period had the highest number of extracted recommendations (954, 21%). Conversely, only 100 (2%) recommendations for the post-discharge phase could be extracted. The most prevalent surgical safety area was ‘Patient support and complication prevention’ with 1471 (31%) recommendations, followed by ‘Preoperative evaluation and planning’ (617, 13%), ‘Standard surgical and anaesthetic procedures for preventing harm’ (468, 10%), and ‘Health care infection prevention’ (462, 10%). The ‘discharge and outpatient follow-up’ recommendations were the least represented (57% and 1% respectively).

**Table 2:** Surgical safety areas, perioperative period, setting and normalized level of evidence, by normalized strength of recommendation

	Weak or not reported (*n* = 2571)	Strong (*n* = 2095)
**Surgical safety area and subarea**		
Diagnosis and referral (*n* = 56)	**28** (**50)**	**28** (**50)**
Delays in the surgical process	14 (51.9)	13 (48.1)
Diagnosis and complication/deterioration rescue	14 (48.3)	15 (51.7)
Preoperative evaluation and planning (*n* = 617)	**330** (**53.5)**	**287** (**46.5)**
Preoperative evaluation and testing	127 (55.5)	102 (44.5)
Preoperative preparation, treatment and prehabilitation	125 (55.8)	99 (44.2)
Anaesthesia and surgical planning	78 (47.6)	86 (52.4)
Patient information and communication (*n* = 236)	**143** (**60.6)**	**93** (**39.4)**
Clear and transparent communication, and patient engagement	75 (62)	46 (38)
Language issues, including health literacy	18 (51.4)	17 (48.6)
Information and Informed Consent	27 (58.7)	19 (41.3)
Postoperative follow up	23 (67.6)	11 (32.4)
Healthcare provider communication and handovers (*n* = 193)	**89** (**46.1)**	**104** (**53.9)**
Surgical safety checklist	30 (62.5)	18 (37.5)
Verbal instructions management	2 (10)	18 (90)
Handovers	34 (50.7)	33 (49.3)
Teamwork and human factor issues	23 (39.7)	35 (60.3)
Monitoring and registries (*n* = 255)	**179** (**70.2)**	**76** (**29.8)**
Intra- and postoperative monitoring	144 (70.6)	60 (29.4)
Clinical records	35 (68.6)	16 (31.4)
Patient support and complication prevention (*n* = 1471)	**819** (**55.7)**	**652** (**44.3)**
Wrong surgery/side/patient	25 (83.3)	5 (16.7)
Hypothermia	24 (42.9)	32 (57.1)
Airway management	12 (30)	28 (70)
Bleeding and transfusion, including PBM	193 (61.3)	122 (38.7)
Anaphylaxis	9 (69.2)	4 (30.8)
Intraoperative awareness	8 (88.9)	1 (11.1)
Malignant hyperthermia	5 (100)	0 (0)
Retained foreign body	1 (100)	0 (0)
Fire on the patient	17 (37.8)	28 (62.2)
Pain	109 (65.7)	57 (34.3)
Nausea and vomiting	25 (52.1)	23 (47.9)
Postoperative delirium and cognitive dysfunction	32 (62.7)	19 (37.3)
Postoperative thromboembolism	57 (43.2)	75 (56.8)
Postoperative myocardial infarction	15 (51.7)	14 (48.3)
Falls	4 (44.4)	5 (55.6)
Pressure ulcers	8 (80)	2 (20)
Other	276 (53.9)	236 (46.1)
Standard surgical and anaesthetic procedures for preventing harm (*n* = 468)	**266** (**56.8)**	**202** (**43.2)**
Safe medication use (*n* = 170)	**115** (**67.6)**	**55** (**32.4)**
Safe blood derivates management (*n* = 39)	**14** (**35.9)**	**25** (**64.1)**
Healthcare infection prevention (*n* = 462)	**250** (**54.1)**	**212** (**45.9)**
Surgical site postoperative infection or/and abscess	189 (50.8)	183 (49.2)
Pneumonia	5 (100)	0 (0)
Bacteremia	21 (77.8)	6 (22.2)
Urinary infection	14 (58.3)	10 (41.7)
Mixed	12 (57.1)	9 (42.9)
Other	10 (76.9)	3 (23.1)
Safe equipment and set up (*n* = 235)	**135** (**57.4)**	**100** (**42.6)**
Equipment maintenance	14 (53.8)	12 (46.2)
Surgical and anaesthetic equipment set up	121 (57.9)	88 (42.1)
Safety structures (*n* = 90)	**24** (**26.7)**	**66** (**73.3)**
Quality and patient safety team	5 (21.7)	18 (78.3)
Incident reporting systems	7 (38.9)	11 (61.1)
Serious adverse events or sentinel events system	5 (50)	5 (50)
Safety rounds	0 (0)	4 (100)
Prospective risk analysis	7 (20)	28 (80)
Human resources (*n* = 258)	**103** (**39.9)**	**155** (**60.1)**
Patient safety training	28 (51.9)	26 (48.1)
Professional training	56 (36.4)	98 (63.6)
Staffing levels	19 (38)	31 (62)
Safety evaluation (*n* = 59)	**30** (**50.8)**	**29** (**49.2)**
Patient safety reports	14 (46.7)	16 (53.3)
Patient safety indicators	16 (55.2)	13 (44.8)
Discharge and outpatient follow-up (*n* = 57)	**44** (**77.2)**	**13** (**22.8)**
**Perioperative phase**		
Preadmission	272 (10.6)	253 (12.1)
Preoperative	414 (16.1)	287 (13.7)
Intraoperative	454 (17.6)	500 (23.9)
Postoperative	504 (19.6)	218 (10.4)
Post-discharge	71 (2.8)	29 (1.4)
* *Mixed	856 (33.3)	808 (38.5)
**Clinical setting**		
Inpatient	992 (38.5)	910 (43.5)
Outpatient	92 (3.6)	87 (4.2)
Both	1487 (57.9)	1 098 (52.4)
**Normalized level of evidence**		
A	73 (2.8)	322 (15.4)
B	212 (8.3)	413 (19.7)
C	348 (13.6)	330 (15.7)
D	271 (10.6)	840 (40.1)
Not reported	1 667 (64.8)	190 (9.2)

Values are *n* (%). Bold values refer to surgical safety areas. Non-bold values refer to surgical safety subareas. PBM, patient blood management.

Of the 4666 retrieved recommendations, 2095 (45%) were strongly recommended based on their normalized SR (*Table 2*). ‘Patient support and complication prevention’, ‘Perioperative evaluation and planning’, ‘Health care infection prevention’, and ‘Standard surgical and anaesthetic procedures for preventing harm’ accounted for most of the strong recommendations (31%, 14%, 10%, and 10% of the strong recommendations respectively).

Overall, the comparative percentage of strong recommendations by area was balanced, although some areas such as ‘Safety structures’, ‘Safe blood derivates management’, and ‘Human resources’ had a higher percentage of strong recommendations (all three over 60% of strong recommendations) whereas ‘Patient information and communication’, ‘Safe medication use’, ‘Monitoring and registries’, and ‘Discharge and outpatient follow-up’ showed lower percentages of strong recommendations (between 39% and 23% of strong recommendations in each category).

Level of evidence supporting strong and not strong recommendations was not equally distributed (χ^2^ with any *P* < 0.001; *Table 2*). Although ‘A’ LE was slightly more prevalent in strong recommendations (15% *versus* 6%), almost two-thirds of the strong recommendations came from normalized LE lower than ‘B’, where the ‘D’ level accounted for 40% (*Table 2*). In a subgroup analysis on strong recommendations, ‘C’ and ‘D’ LE were independently associated with higher rigour of development AGREE-II scores than ‘A’, ‘B’, or ‘Not reported’, with a statistically significant difference greater than 8.7% (4.90–12.59%) in each pairwise comparison (any *P* < 0.001, using Tukey’s adjustment for multiple comparisons).

‘Health care infection prevention’, ‘Discharge and outpatient follow-up’, and ‘Safe blood derivates management’ were the three surgical safety areas with the highest percentage of LE ‘A’ (from 21 to 13%).

### Methodological quality of clinical practice guidelines containing strong recommendations

Clinical practice guidelines containing at least one strong recommendation accounted for one-third of the documents included in this review (94, 35%; *Table 1*). Of these, 89% reported a structured and detailed evidence review, 98% used an explicit grading or consensus method, and 65% were reviewed using an external panel. Their median ‘rigour of development’ score was 53% (i.q.r. 36–67%), and only 18 were of ‘high quality’, as presented in *Table 3*. Seven were pathology-oriented or related to specific surgical or anaesthetic approaches^34,35,37,40,47–49^, nine were addressed to prevent or manage specific complications^19,29,36,38,39,41–44^, and two were general guidelines for any surgical patient^33,46^.

**Table 3:** Detailed description of high-quality clinical practice guidelines with at least a ‘Strong’ recommendation

Authors	Title	Method for grading recommendations	AGREE-II domain 3 score	Scope	Original language
AAOS^34^	Clinical practice guideline on the management of osteoarthritis of the hip	AAOS (2017)	83.3	National	English
AAOS^35^	Management of hip fractures in older adults	AAOS (2017)	81.3	National	English
AGS^36^	American Geriatrics Society abstracted clinical practice guideline for postoperative delirium in older adults	ACP (2010)	72.9	National	English
Anne *et al*.^37^	Clinical practice guideline: opioid prescribing for analgesia after common otolaryngology operations	Adapted from Oxford CEBM	84.4	International	English
AORN^33^	Guidelines for perioperative practice	AORN (2022)	79.2	International	English
Apfelbaum *et al*.^38^	2022 American Society of Anesthesiologists practice guidelines for management of the difficult airway	ASA (2016)	71.9	International	English
Berríos-Torres *et al*.^39^	Centers for Disease Control and Prevention guideline for the prevention of surgical site infection, 2017	Adapted from GRADE	76.0	National	English
Boselli *et al*.^40^	European Society of Anaesthesiology guidelines on peri-operative use of ultrasound for regional anaesthesia (PERSEUS regional anesthesia)	Adapted from GRADE	82.3	International	English
Brunt *et al*.^41^	Safe cholecystectomy multi-society practice guideline and state of the art consensus conference on prevention of bile duct injury during cholecystectomy	GRADE	91.7	International	English
Department of Health^42^	Nutrition screening and use of oral nutrition support for adults in the acute care setting	SIGN (2019) and GRADE	87.5	National	English
Devlin *et al*.^43^	Clinical practice guidelines for the prevention and management of pain, agitation/sedation, delirium, immobility, and sleep disruption in adult patients in the ICU	GRADE	81.3	National	English
IMSS^44^	Prevention and management of complications postoperative in non-cardiac surgery in the older adult	Shekelle *et al*.^45^, SIGN (2012) and GRADE	70.8	National	Spanish
IMSS^46^	Preventive interventions for safety in the surgical patient	Shekelle *et al*.^45^ and GRADE	74.0	National	Spanish
Korytkowski *et al*.^29^	Management of hyperglycemia in hospitalized adult patients in non-critical care settings: an Endocrine Society clinical practice guideline	GRADE	84.4	International	English
Murray *et al*.^47^	Clinical practice guidelines for sustained neuromuscular blockade in the adult critically ill patient	GRADE	70.8	International	English
Nast *et al*.^48^	S3 guideline: management of anticoagulants and antiplatelet agents in cutaneous surgery	GRADE	74.0	National	English
RNAO^49^	Supporting adults who anticipate or live with an ostomy	GRADE	100.0	National	English
WHO^19^	Global guidelines for the prevention of surgical site infection	GRADE	80.2	International	English

AAOS, American Academy of Orthopaedic Surgeons; ACP, American College of Physicians; AGREE-II, Appraisal of Guidelines for Research and Evaluation II; AGS, American Geriatrics Society; AORN, Association of periOperative Registered Nurses; ASA, American Society of Anesthesiologists; CEBM, Center for Evidence-Based Medicine; GRADE, Grading of Recommendations, Assessment, Development, and Evaluations; IMSS, Instituto Mexicano del Seguro Social; RNAO, Registered Nurses’ Association of Ontario; SIGN, Scottish Intercollegiate Guidelines Network.

Most CPGs containing strong recommendations provided additional tools or materials for implementation support (67, 71%), with a close prevalence in those classified as ‘High quality’ (12, 67%). These materials were heterogeneous in type and content, ranging from management algorithms, scales for diagnosis or screening, and other clinical resources, to educational and infographic materials for both patients and professionals. Conversely, the number of CPGs containing performance indicators was below 17% in both groups. No statistically significant differences were found between the groups. The list of references of the 12 high-quality CPGs containing tools or materials for supporting their implementation and the three high-quality CPGs containing indicators are provided in *Table S1*.

### Strong recommendations for perioperative patient safety in high-quality clinical practice guidelines

A subset of 18 high-quality CPGs provided 828 extracted recommendations, of which 562 (68%) had a ‘Strong’ SR that could be regarded as priority recommendations. *Table S2* describes their distribution in phases of the perioperative care continuum and the level of evidence by safety area.

The most prevalent topics included anaesthesia and ultrasound-guided procedures, team roles and perioperative team approaches, and safety practices related to sterile fields, skin antiseptics, hand hygiene, medication administration, and surgical item counting and control. Although many recommendations were distributed throughout all periods of the perioperative care continuum, the intraoperative period and the area related to complication prevention were the most populated separately and together, and only 30 (5%) were identified as applicable to the out-of-hospital phases (that is pre-admission or post-discharge).

The final combination of ‘high-quality’ CPG, ‘Strong’ SR, and ‘A’ normalized LE yielded 78 recommendations, as listed in *Table 4*.

**Table 4:** Verbatim description of the recommendations with ‘Strong’ grading and ‘A’ level of evidence from high-quality clinical practice guidelines

Patient safety area	Patient safety subarea (if any)	Verbatim description of the recommendation
**Preadmission**		
Preoperative evaluation and planning	Preoperative evaluation and testing	Prior to surgery, clinicians should identify risk factors for Opioid Use Disorder when analgesia using opioids is anticipated^37^.
Patient information and communication	Including patient engagement and transparency	Provide clear verbal and written instructions for preoperative bathing to the patient and patient care provider^33^.
Healthcare infection prevention	Surgical site Postoperative Infection or/and abscess	It is recommended that mechanical bowel preparation not be used routinely to prevent SSI^46^.
		It is recommended that patients take a soapy bath at least the night before surgery^46^.
		Patients should complete preoperative bathing at least once on the night before or the day of the operative or other invasive procedures^33^.
		Instruct patients undergoing procedures of the head or neck to shampoo their hair before surgery^33^.
**Preoperative**		
Patient support and complication prevention	Hypothermia	When active warming is indicated, prewarm the patient with the selected method^33^.
	Bleeding and transfusion, including PBM	In healthy patients undergoing major elective surgery, erythropoietin can be used in combination with autologous blood donation or to obtain multiple red cell donations and maintain adequate Hb on the day of surgery^46^.
		Erythropoietin should be given to patients under 70 years of age scheduled for surgery with major blood loss and Hb < 13 g/dl^46^.
	Anaphylaxis	Assess the patient for allergies and sensitivities to preoperative skin antiseptics before selecting the antiseptic^33^.
	Pain	Multimodal analgesia incorporating preoperative nerve block is recommended to treat pain after hip fracture^35^.
Standard surgical and anaesthetic procedures	Standard surgical and anaesthetic procedures	Preoperative traction should not routinely be used for patients with a hip fracture^35^.
Healthcare infection prevention	Surgical site postoperative infection or/and abscess	Administer the appropriate parenteral prophylactic antimicrobial agents before skin incision in all caesarean section procedures^39^.
		After the preoperative bath or shower, instruct the patient not to apply alcohol-based hair or skin products, deodorant (when the axilla will be in the sterile field), lotions, emollients, or cosmetics^33^.
		If indicated, remove hair at the surgical site by clipping or depilatory methods in a manner that minimizes injury to the skin^33^.
		When it is necessary to shave, it is recommended to use an electric razor with a single-use head on the same day of surgery. The use of razor blades for shaving is not recommended because they increase the risk of SSI^46^.
		When removing hair outside the OR or procedure room is not possible, remove the patient’s hair in a manner that prevents dispersal of hair into the air of the OR or procedure room (for example wet clipping, use of a vacuum device)^33^.
		Nasal decontamination with topical antimicrobial agents to eliminate *Staphylococcus aureus* is not routinely recommended to reduce the risk of SSI^46^.
		When hair removal is indicated, the amount of hair removed should be kept to a minimum^33^.
		When hair removal is indicated, remove hair as close to the start of surgery as feasible in a location outside the OR or procedure room^33^.
		Leave hair at the surgical site in place unless hair removal is indicated^33^.
		Develop a standardized protocol for preoperative bathing that includes dose (volume or amount of the product), frequency (number of applications), and duration (exposure time of skin to the antiseptic)^33^.
**Intraoperative**		
Preoperative evaluation and planning	Anaesthesia and surgical planning	Either spinal or general anaesthesia is appropriate for patients with a hip fracture^35^.
Healthcare provider communication and handovers	Surgical safety checklist	Use a standardized surgical safety checklist during the time-out process^33^.
Monitoring and registries	Intra- and postoperative monitoring	Monitor the amount of fluid dispensed and collected during the procedure in collaboration with the anaesthesia professional^33^.
Patient support and complication prevention	Bleeding and transfusion, including PBM	Tranexamic acid can be used to reduce blood loss and transfusion requirements in patients scheduled for knee replacement surgery when other blood-sparing techniques are inappropriate and increased blood loss is anticipated^46^.
		The use of tranexamic acid is recommended in patients undergoing elective cardiac surgery at high risk of transfusion^46^.
		Tranexamic acid should be administered to reduce blood loss and blood transfusion in patients with hip fractures^35^.
	Fire on the patient	Identify potential hazards associated with fire safety and establish safe practices for communication, prevention, suppression, and evacuation^33^.
	Other	Position patients in the prone position in 5-degree to 10-degree reverse Trendelenburg, if possible^33^.
		Position a pregnant woman undergoing obstetric surgery in a left lateral tilt by placing a 4.7-inch (12-cm) wedge-shaped positioning device under the right lumbar region above the iliac crest and below the lower costal region to achieve a 12-degree to 15-degree lateral tilt, placing a wedge-shaped positioning device under the right pelvis to achieve a 12-degree to 15-degree lateral tilt, or tilting the OR bed 15 degrees to 45 degrees to the left^33^.
		Evacuate and filter all surgical smoke^33^.
		Maintain insufflation pressure at the lowest level necessary to achieve pneumoperitoneum within the specification of the surgeon^33^.
Standard surgical and anaesthetic procedures	Standard surgical and anaesthetic procedures	Implement safe practices when positioning the patient in the reverse Trendelenburg or modifications of the reverse Trendelenburg position^33^.
		In patients with unstable (displaced) femoral neck fractures, arthroplasty is recommended over fixation^35^.
		In patients undergoing arthroplasty for femoral neck fractures, the use of cemented femoral stems is recommended^35^.
Healthcare infection prevention	Surgical site postoperative infection or/and abscess	Perform intraoperative skin preparation with an alcohol-based antiseptic agent unless contraindicated^39^.
		In clean and clean-contaminated procedures, do not administer additional prophylactic antimicrobial agent doses after the surgical incision is closed in the operating room, even in the presence of a drain^39^.
		In prosthetic joint arthroplasty, if clean and clean-contaminated procedures, do not administer additional prophylactic antimicrobial agent doses after the surgical incision is closed in the operating room, even in the presence of a drain^39^.
		It is recommended not to routinely use self-adhesive surgical drapes as they may increase the risk of SSI^46^.
		Include the following in isolation technique procedures: organizing the sterile field in a manner that minimizes the risk of sterile field exposure to intestinal tract bacteria or cancerous cells from metastatic tumour excisions; initiating isolation technique immediately before resection of the bowel or metastatic tumour and concluding when the resection or anastomosis is complete; no longer using instruments or items that had contact with the inside of the bowel lumen after it has been closed or that were used for metastatic tumour excision; removing contaminated instruments and items from the sterile field or placing them in a separate area that will not be touched by members of the sterile team; changing surgical gloves and changing the surgical gown when soiled; covering existing sterile drapes with new sterile drapes; and using clean instruments to close the wound after anastomosis or resection^33^.
		When open sterile supplies are present, wear a clean surgical mask that covers the mouth and nose and is secured in a manner that prevents venting at the sides of the mask^33^.
		Do not use adhesive incise drapes without antimicrobial properties^33^.
		Immediately before presenting items to the sterile field, inspect sterile items for sterility of the contents, as noted on the packaging; the expiration date, when applicable; package integrity; product integrity (for example discoloration or particulate formation in medications and solutions); and verification that the external chemical indicators have changed to the correct colour, indicating that the parameters for sterilization have been met^33^.
		Introduce sterile items to the sterile field as close as possible to the time of use^33^.
		Prepare the sterile field as close as possible to the time of use^33^.
		Select the antiseptic product based on the anatomical location of the surgical procedure^33^.
		Change surgical gloves worn during invasive surgical procedures: after each patient procedure; every 90–150 min; when a visible defect or perforation is noted or when a suspected or actual perforation from a needle, suture, bone, or other object occurs; immediately after direct contact with methyl methacrylate; after touching optic eye pieces on the operative microscope; after touching a fluoroscopy machine; after touching a surgical helmet system hood or visor; and when suspected or actual contamination occurs^33^.
		Select an alcohol-based skin antiseptic for surgical site preparation unless contraindicated^33^.
		When a unidirectional ultraclean air delivery system (for example laminar airflow) is in use, position the surgical site and instrument tables within the air curtain of the system, if possible^33^.
		Use sterile technique when donning, wearing, and changing sterile gloves^33^.
		Scrubbed team members should wear two pairs of sterile surgical gloves (that is, double glove), and use a perforation indicator system^33^.
		Do not apply microbial sealant after surgical skin preparation^33^.
Safety structures	Prospective risk analysis	Identify potential patient injuries and complications associated with gas insufflation media used during minimally invasive surgical procedures and establish practices that reduce the risk for injuries and complications^33^.
Human resources	Patient safety training	Incorporate the safe surgery checklist in perioperative team training^33^.
**Postoperative**		
Preoperative evaluation and planning	Patient preparation, including preoperative treatments and prehabilitation	Restoration to sinus rhythm is recommended in patients who develop postoperative atrial fibrillation. In the haemodynamically stable patient, pharmacological cardioversion is recommended (propafenone if there is no structural heart disease or amiodarone if there is structural heart disease), in case of haemodynamic instability, electrical cardioversion^44^.
Patient support and complication prevention	Postoperative thromboembolism/deep venous thrombosis	In all patients who are going to undergo surgery and are going to remain in the hospital after surgery, compression stockings are recommended, preferably those graduated to the hip or thigh, if there is no contraindication^46^.
		Every patient who is going to undergo surgery and is going to remain in the hospital after surgery must receive some effective method of preventing thrombotic complications^46^.
	Other	Chewing gum may be recommended as oral stimulation may act as a mimetic of the feeding process, increasing vagal tone and promoting bowel function. It is a cheap and accessible option for the treatment of postoperative ileus^44^.
		Healthcare professionals should consider giving postabdominal surgery patients who can swallow safely, and in whom there are no specific concerns about gut function or integrity, some oral intake within 24 h of surgery. The patient should be monitored carefully for any signs of nausea or vomiting^42^.
Standard surgical and anaesthetic procedures	Standard surgical and anaesthetic procedures	In the elderly patient with pulmonary thromboembolism in the postoperative period, it is recommended to start anticoagulation with low-molecular-weight heparin at a full dose (Enoxaparin 1 mg/kg/12 h), always considering the risk of bleeding that the type of surgery represents (see surgeries with higher risk of bleeding mentioned above) and absence of kidney failure. It should be started at the same time with a vitamin K antagonist^44^.
**Post-discharge**		
Standard surgical and anaesthetic procedures	Standard surgical and anaesthetic procedures	At the patient’s discharge, the implementation of a muscle stretching programme with progressive resistance 2–3 times a week with a gradual progression in the intensity of the exercise (to the patient’s tolerance) is recommended, as it is an effective intervention to improve functionality in the older adult with some degree of functional impairment^44^.
**Mixed**		
Healthcare provider communication and handovers	Handovers	Take precautions to mitigate the risk for errors during the transitions of care between phases of perioperative care^33^.
Patient support and complication prevention	Hypothermia	Use the same site and method of temperature measurement throughout the perioperative phases when clinically feasible^33^.
		Select the temperature measurement site and method in collaboration with the perioperative team based on the requirements of the procedure, anaesthesia type, anaesthesia delivery method, accessibility of the body site for measurement, and invasiveness of the method^33^.
	Pain	Strong evidence supports that NSAIDs improve short-term pain, function, or both in patients with symptomatic osteoarthritis of the hip^34^.
Standard surgical and anaesthetic procedures	Standard surgical and anaesthetic procedures	Use a risk-based approach that includes local epidemiology, procedure-specific risk factors, and patient risk factors when determining *S. aureus* decolonization strategies^33^.
Safe medication use	Safe medication use	The multidisciplinary team should select technological devices (for example barcode systems, computerized prescriber order entry system, biometrics, pharmacy automation, radiofrequency identification systems, electronic medication storage and inventory systems, electronic medication administration records, electronic medication reconciliation tools) to be used during all phases of the medication use process based primarily on the safety aspects incorporated into each device^33^.
		Involve pharmacists in all phases of medication management^33^.
		Develop and use a double-checking system (for example independent double check) performed by two licensed individuals for predetermined high-alert and high-risk medications (for example insulin, heparin)^33^.
Healthcare infection prevention	Surgical site postoperative infection or/and abscess	It is not recommended to administer insulin routinely in non-diabetic patients to optimize blood glucose in the postoperative period to reduce the risk of SSI. Consider the high risk of hypoglycaemia associated with this intervention^46^.
		Implement perioperative glycaemic control and use blood glucose target levels less than 200 mg/dl in patients with and without diabetes^39^.
		For patients with normal pulmonary function undergoing general anaesthesia with endotracheal intubation, administer increased FiO_2_ intraoperatively and postextubation in the immediate postoperative period. To optimize tissue oxygen delivery, maintain perioperative normothermia and adequate volume replacement^39^.
		Maintain perioperative normothermia^39^.
Safety structures	Safety rounds	Interdisciplinary care programmes should be used in the care of hip fracture patients to decrease complications and improve outcomes^35^.
	Prospective risk analysis	Identify potential injuries and complications associated with fluid used for irrigation or as distension media during minimally invasive surgery and computer-assisted procedures^33^.
Human resources	Patient safety training	Include simulation scenarios that incorporate the healthcare organization’s standardized communication tools for briefing, time out, debriefing, and hand-overs in perioperative team training^33^.

NSAIDs, non-steroidal anti-inflammatory drugs; OR, operating room; PBM, patient blood management; SSI, surgical site infection.

## Discussion

This is the first systematic review of guidelines to comprehensively summarize evidence-based perioperative patient safety recommendations published in the international, national, and regional guidelines. A total of 4086 perioperative patient safety recommendations were extracted from 267 CPGs, position statements, expert consensus, and other types of grey literature from 2012 to 2022. An extensive array of recommendations for perioperative patient safety in the literature was found. Less than half could be catalogued as strongly recommended. Of these, almost two-thirds were supported by low or very low LE, and only 18 guidelines containing strong recommendations had a high rigour of development in their appraisal. This selection of 18 guidelines prompted 562 recommendations, 78 of which had a normalized LE that could eventually be used to define an evidence-based reference framework for patient safety practices.

The distribution of strong recommendations across perioperative care continuum phases in this review was uneven. In particular, there was a remarkable absence of ‘Diagnosis and referral’ and ‘Discharge and outpatient follow-up’ recommendations, which was aligned with the overall scarcity of out-of-hospital recommendations; this denotes a hospital-centred scope in the literature, despite the known relevance of adverse events after discharge from hospital^50,51^. Surgical safety extends beyond the operating room, starting weeks or months before, and continuing after hospital discharge^52,53^. These periods are critical not only because of numerous care transitions, which are prone to failures^54^, but also because of the prevalence of errors, between 53% and 70%, outside the operating room^55^. Thus, enhancing surgical safety requires focusing on the entire surgical pathway using a multidisciplinary approach^56^.

The included guidelines were heterogeneous, with large variations in their scope. Only a few studies have specifically focused on surgical patient safety as a topic, a scarcity in the literature already pointed out by other authors^57,58^, and most of them partially because of their intended focus on single complications or clinical procedures. Conversely, most of these studies were published in high-income countries, which may limit their generalizability and applicability in low- and middle-income countries owing to differences in healthcare infrastructure, resource availability, policy involvement, and cultural factors^59,60^. Additionally, many of these studies had flaws concerning a clear structured review of evidence, an explicit method for grading LE and SR, and an external review.

When developing guidelines for improving safety practices, methodology has been highlighted as the most critical element^58^. The association between strong recommendations supported by low and very low LE and high-quality guidelines suggests that the more rigorous the guideline methodology is, the lower the LE addressed to the safety recommendations. Moderate- and high-quality guidelines perform further and deeper appraisals (for example using GRADE) than low-quality guidelines and identify a higher number of strong recommendations with low and very low LE. This a priori discordant result has also appeared when analysing other guidelines^61^ where recommendations supported by low or very low evidence became strong recommendations if they had a benefit perceived by the panel^62^. The shortage of RCTs and meta-analysis supporting patient safety recommendations may be explained by ethical, technical, and resource reasons: first, there may be ethical concerns in studying the potential benefits of an already strong safety recommendation through an RCT, despite it being issued based on low LE. Second, measuring patient safety is difficult considering that the main outcome is not the positive impact of a clinical intervention on patient outcomes, but the potential avoidance of the negative impact of adverse events and the mitigation of risks^63^. Third, despite the growing recognition of the costs of patient safety issues^64^, patient safety research funding lacks the incentive of other industry-driven initiatives.

The recommendations extracted provide a support to systematically improve safety, not just as the absence of patient harm, but through the improvement of safety culture, education, adherence to quality improvement cycles, and patient engagement. Additionally, the high-quality CPGs gathered in this study may provide extremely valuable inputs and resources for stakeholders, managers, and policymakers that facilitate the enactment and adoption of good practices, namely those that have accounted for materials for implementation during the implementation process^65^. The tools, materials, and quality indicators identified in this review and available as *Supplementary materials* may be particularly useful for the improvement teams^66–68^. They may reduce the burden of implementation by adapting to local needs, as this has been successfully demonstrated as a key factor in implementing surgical safety checklists^69,70^. The same rationale has been promoted by the ADAPTE initiative for adapting developed guidelines to diverse local contexts^71^, enhancing their applicability and adherence to evidence through acceptability and a sense of ownership by end-users, while avoiding duplication of work.

This study had some limitations. First, a qualitative analysis is not performed for the selected recommendations. The quality of the wording may be limited, and some repetition and overlapping of concepts may be present in the practices for enhancing the perioperative safety provided in this study. This systematic review may serve as the basis for subsequent conceptual revisions, syntheses, and consensus methodologies, which would be beneficial for defining more robust recommendations. However, the strength of the selection of recommendations has been based on scientific, albeit practical, criteria that prioritize the best methodological recommendations with a greater healthcare impact.

In this regard, any publication and recommendation regardless of its methodological quality or LE were included and, instead, SR was prioritized as the main criterion. As discussed before, LE does not strictly represent the balance of benefits and harms^72,73^ and may not capture the full picture of preventive patient safety recommended interventions^73^. As the focus was on the highest SR, the selected recommendations should represent those with a higher positive risk–benefit ratio integrating the patient safety evidence limitations.

Second, an ad-hoc algorithm to harmonize the levels of evidence that have not been validated was developed. Many authors have stressed the heterogeneity of evidence grading systems in the international body of guidelines, and the problems associated with this matter^74–77^. Given that GRADE is probably the most used and robust grading system, in the absence of a well-established method for this harmonization, this algorithm is envisioned as a pragmatic solution for approximating them to GRADE. Although this classification system was based on proxies, it is explicitly described and could be useful in further research for translating among the most prevalent grading systems, wherever the most accurate quality of evidence grading is not needed.

Third, acknowledging the need for rigorous quality assessment^78^, every guideline was appraised using three fundamental methodological criteria, focusing on a deeper methodological appraisal of a restricted group of guidelines. The third domain (rigour of development) of AGREE-II was applied, as it may have the strongest correlation with the overall guideline quality^79^. These results are similar to those of previous systematic reviews^80^. Although these findings demonstrate a wealth of recommendations available to inform perioperative patient safety, they also reflect the necessity for the standardized development of high-quality guidelines in the field.

This study offers a comprehensive analysis of the key determinants of evidence-based practice adoption in the patient safety field. The selected 78 recommendations, characterized by their high level of evidence, strong recommendation, and high-quality CPG, are essential for implementing scientifically validated practices. However, patient safety experts, clinicians, and patient representatives may perceive these recommendations, although solidly founded, as incomplete. Considering the remaining strong recommendations could present valuable opportunities for further improving patient outcomes.

Future research should focus on the following areas: reduce the discordance between SR and LE through dedicated research; fill the gaps identified in areas with few strong recommendations in the perioperative period; synthesize and prioritize the existing recommendations; apply implementation science to understand how to integrate recommendations into diverse healthcare systems, including low-resource settings; and analyse potential barriers and facilitators to the implementation of recommendations.

This review adds to previous evidence a comprehensive patient safety-focused compilation and analysis of guidelines across a broad range of perioperative care aspects, revealing a disparity in the characteristics and methodological quality of the guidelines, as well as in the strength and evidence level of recommendations. It also highlights the available strongly recommended, evidence-based recommendations found in high-quality CPGs, and some associated implementation tools. The selected guidelines and recommendations may be useful through contextual adaptation and implementation to improve patient safety in perioperative care at both the unit and hospital levels, as well as in developing general policies at national and international levels. Finally, with the discussed limitations, the areas of discordance between SR and LE may be prioritized for future research to facilitate resource allocation for their implementation and frontline providers’ adherence.

## Collaborators

Joaquim Baneres, Genis Carrasco, Rosa Sunol and Helena Vall (Avedis Donabedian Research Institute (FAD), Barcelona, Spain); Hiske Calsbeek, Yvette Emond and Anita Heideveld-Chevalking (Radboud University Medical Center, IQ Health Scientific Department, OR Department, Nijmegen, The Netherlands); Pedro Casaca-Carvalho, Andreia Leite, Ana Beatriz Nunes and Ayshe Seyfulayeva (NOVA National School of Public Health, Comprehensive Health Research Center, CHRC, NOVA University Lisbon, Lisbon, Portugal); Edoardo De Robertis, Cathy Weynants and Maria Wittman (European Society of Anaesthesiology and Intensive Care (ESAIC), Brussels, Belgium); Pascal Garel and Marie Nabbe (European Hospital and Healthcare Federation (HOPE), Brussels, Belgium); Oliver Groene and Sophie Wang (OptiMedis AG, Hamburg, Germany); Mari Kangasniemi, Janne Kommusaar, Kaja Kristensen, Kaja Polluste, Janne Pühvel and Joel Starkopf (University of Tartu, Tartu, Estonia); David Marx and Frantisek Vlcek (Spojená akreditační komise, Prague, Czech Republic); Willemijn Schäfer, Caroline Schlinkert, Nina van der Schoot, Lilian Van Tuyl, Marieke Voshaar and Cordula Wagner (Netherlands Institute for Health Services Research (NIVEL), Utrecht, The Netherlands).

## Supplementary Material

zrae143_Supplementary_Data

## Data Availability

Supplementary materials are provided with the data used in this systematic review. Other data files are available under reasonable request.
