# Supplementary material for: Perioperative patient safety recommendations: systematic review of clinical practice guidelines
Source: BJS Open. 2024 Dec 11;8(6):zrae143. doi: 10.1093/bjsopen/zrae143 (PMC11632830; doi:10.1093/bjsopen/zrae143)
Supplement: zrae143_Supplementary_Data [file zrae143_supplementary_data.docx]

**Title**

**Perioperative Patient Safety Recommendations: Systematic Review of Clinical Practice Guidelines**

Authors

Ismael Martínez-Nicolas^1^, PhD; Daniel Arnal-Velasco^1,2^, MD; Eva Romero-Garcia^1,3^, MD; Neus Fabregas^1,4^, MD, PhD; Yolanda Sanduende-Otero^1,5^, MD; Irene Leon^1,4^, PhD; Ashish A. Bartakke^1,6^, MD; Javier Silva-Garcia^1,7^, MD; Anna Rodriguez^8,9^, PhD; Claudia Valli^8,9^, PhD; Sandro Zamarian^10^, MD; Adam Zaludek^10,11^, PhD; Jose Meneses-Echavez^12^, PhD; Andrés F. Loaiza-Betancur^13^, MSc; Paulo Sousa PhD^14^, MD; Carola Orrego^8,9,15^, PhD; Victor Soria-Aledo^1,16^, PhD; on behalf of SAFEST Consortium.

^1^Spanish Anaesthesia and Reanimation Incident Reporting System (SENSAR), Alcorcon, Spain.

^2^Department of Anaesthesia and Reanimation. Hospital Universitario Fundación Alcorcon. Alcorcon. Spain.

^3^Department of Anaesthesia and Reanimation. Hospital Univesitari Policilic La Fe. Valencia. Spain.

^4^Department of Anaesthesia and Reanimation. Hospital Clinic. Barcelona. Spain.

^5^Department of Anaesthesia and Reanimation. Hospital Universitario de Pontevedra. Pontevedra. Spain.

^6^Department of Anaesthesia and Reanimation, Hospital Valle de Los Pedroches. Pozoblanco, Spain.

^7^Department of Anaesthesia and Reanimation, Hospital Universitario 12 de Octubre. Madrid, Spain.

^8^Avedis Donabedian Research Institute, Barcelona, Spain.

^9^Universidad Autónoma de Barcelona, Spain.

^10^Spojená Akreditační Komise. Prague. Czech Republic.

^11^Charles University, Third Faculty of Medicine, Department of Public Health. Prague. Czech Republic.

^12^Norwegian Institute of Public Health. Oslo. Norway.

^13^Instituto Universitario de Educación Física. Universidad de Antioquia. Medellín, Colombia.

^14^NOVA National School of Public Health, Comprehensive Health Research Center, CHRC, NOVA University, Lisbon, Portugal.

^15^Network for Research on Chronicity, Primary Care, and Health Promotion (RICAPPS). Barcelona, Spain.

^16^Department of surgery, Hospital Universitario Morales Meseguer. Murcia. Spain.

**Collaborators:**

SAFEST Consortium: Daniel Arnal-Velasco^1^, Joaquim Baneres^2^, Ashish Bartakke^1^, Hiske Calsbeek^3^, Genis Carrasco^2^, Pedro Casaca-Carvalho^4^, Edoardo De Robertis^5^, Yvette Emond^3^, Neus Fabregas^1^, Javier Silva-García^1^, Pascal Garel^6^, Oliver Groene^7^, Anita Heideveld-Chevalking^3^, Mari Kangasniemi^8^, Janne Kommusaar^8^, Kaja Kristensen^8^, Andreia Leite^4^, Irene Leon^1^, Ismael Martínez-Nicolás^1^, David Marx^9^, Marie Nabbe^6^, Ana Beatriz Nunes^4^, Carola Orrego^2^, Kaja Polluste^8^, Janne Pühvel^8^, Eva Romero-Garcia^1^, Yolanda Sanduende-Otero^1^, Willemijn Schäfer^10^, Caroline Schlinkert^10^, Ayshe Seyfulayeva^4^, Victor Soria-Aledo^1^, Paulo Sousa^4^, Joel Starkopf^8^, Rosa Sunol^2^, Helena Vall^2^, Claudia Valli^2^, Nina van der Schoot^10^, Lilian Van Tuyl^10^, Frantisek Vlcek^9^, Marieke Voshaar^10^, Cordula Wagner^10^, Sophie Wang^7^, Adam Žaludek^9^, Sandro Zamarian^9^.

^1^Spanish Anaesthesia and Reanimation Incident Reporting System (SENSAR), Alcorcon, Spain

^2^Avedis Donabedian Research Institute (FAD), Barcelona, Spain

^3^Radboud University Medical Center (IQ Health Scientific Department, OR Department, Nijmegen, The Netherlands

^4^NOVA National School of Public Health, Comprehensive Health Research Center, CHRC, NOVA University Lisbon, Lisbon, Portugal

^5^European Society of Anesthesiology and Intensive Care (ESAIC), Brussels, Belgium

^6^European Hospital and Healthcare Federation (HOPE), Brussels, Belgium

^7^OptiMedis AG, Hamburg, Germany

^8^University of Tartu, Tartu, Estonia

^9^Spojená akreditační komise, Prague, Czech Republic

^10^Netherlands Institute for Health Services Research (NIVEL), Utrecht, The Netherlands

**Corresponding author.** Daniel Arnal-Velasco. [darnal@sensar.org](mailto:darnal@sensar.org). Department of Anaesthesia and Reanimation. Hospital Universitario Fundación Alcorcon. Budapest 1. 28229 Alcorcon. Spain. **ORCID ID 0000-0002-8016-4472**; **Twitter** darnalvel

# Supplementary Materials - Index

| **Supplementary Methods** |  |
| --- | --- |
| M1 Search Question and PICAR Statement | *pag. 3* |
| M2 Surgical patient safety recommendation areas and subareas | *pag. 4* |
| M3 Search Strategy | *pag. 5* |
| M4 Data extraction forms and variables description  M5 Levels of Evidence algorithm. Methodological explanation | *pag. 9*  *pag. 12* |
| **Supplementary Appendixes** |  |
| A1 Prisma 2020 Checklist | *pag. 14* |
| **Supplementary Figures and Tables** |  |
| Table S1. High-quality Clinical Practice Guidelines containing tools or materials for supporting their implementation. | *pag. 18* |
| Table S2. Distribution across perioperative period and level of evidence of the strong recommendations from high-quality clinical practice guidelines (“rigour of development” > 70%) (n=562) | *pag. 21* |
| **References** | *pag. 21* |
|  |  |

# Supplementary Methods

## M1 Search Question and PICAR Statement

**What are the recommendations to improve patient safety in the perioperative period among adults?**

This search aims to answer:

1. What are the recommended patient safety practices in the perioperative care continuum in adults?
2. What is the strength of recommendations and the quality of evidence for these patient safety practices?

Eligibility criteria pertaining to the population and clinical areas, interventions, comparators, attributes of CPGs and recommendation characteristics were detailed following PICAR statement (Table M1.1).

**Table M1.1. PICAR criteria defined in our study**

| PICAR Element | Study Specific Criteria |
| --- | --- |
| Population & Clinical area(s) | - Adult (≥18 years)  - Surgical process |
| Interventions | - **Interventions:** papers that report at least one patient safety recommendation |
| Comparators | No comparator applicable. All recommendations for patient safety will be included. |
| Attributes of eligibility | - **Language:** European Union (EU) & European Economic Area (EEA) languages  **- Publication years:** 2012 to 2022, considering two potential cycles of 5 years maximum updating period for CPGs  - **Type of publication**: Clinical Practice Guidelines (CPG), Position statements, Expert consensus, other individual or grouped recommendations.  - **Publishing region:** All  - **Clinical Scope:** papers primarily focused on the recommendations for the perioperative period in adult patients.  - **Format**: All  - **Specific methodical standards:** All  - **System of rating evidence:** All  - **Version**: latest available. Direct translations or interpretations of other guidelines will be excluded.  - **Purpose:** Reducing preventable morbidity and mortality associated with the surgical process.  - **Interventions:** papers that report at least one recommendation focused on patient safety practices. |
| Recommendation characteristics | - **Scope:** Adult patient safety recommendations  - **Duration of treatment:** Recommendations for the perioperative care continuum*, from the surgical indication up to 90 days after surgery.  - **Levels of confidence:** non-applicable  - **Comparators**: Non-applicable.  - **Locating recommendations**: Texts, tables, algorithms and/or decision paths. |
| *Perioperative care continuum: pre-operative out-of-hospital (preadmission); pre-operative in-hospital; intra-operative in-hospital; post-operative in-hospital; and post-operative out-of-hospital (postdischarge). | |

## M2 Surgical patient safety recommendation areas and subareas

1. Diagnosis and referral.
2. Delays in the surgical process once the surgical indication has been set.
3. Diagnosis of complications during the surgical process. Rescue the deteriorating patient.
4. Preoperative evaluation and planning.
5. Preoperative testing
6. Patient preparation. Including preoperative treatments.
7. Surgical planning.
8. Patient information and communication
9. Including patient engagement and transparency.
10. Language issues.
11. Information and Informed Consent.
12. Postoperative follow up.
13. Health care provider communication and handovers
14. Surgical safe checklist.
15. Verbal instructions.
16. Handovers.
17. Teamwork and human factor issues: leadership, task assignment, situation awareness.
18. Monitoring and registries
19. Intra and postoperative monitoring.
20. Clinical records.
21. Patient support and complication prevention
22. Wrong surgery/wrong side/wrong patient.
23. Hypothermia.
24. Airway management
25. Bleeding
26. Anaphylaxis
27. Intraoperative awareness
28. Malignant hyperthermia
29. Retained foreign object
30. Fire in the patient
31. Pain
32. Nausea and vomiting
33. Surgical or postoperative infection
34. Postoperative delirium and cognitive impairment
35. Postoperative Pneumonia
36. Postoperative catheter infection
37. Postoperative Thromboembolism
38. Postoperative myocardial infarction
39. Standard operation procedures
40. Safe medication use
41. Safe blood derivates management
42. Health care infection prevention
43. Safe Equipment and set up
44. Equipment maintenance
45. Equipment set up
46. Safety structures
47. Quality & Patient safety team
48. Incident reporting systems
49. Serious adverse events or Sentinel events system
50. Safety rounds
51. Prospective risk analysis
52. Human resources
53. Patient safety training
54. Staffing levels (e.g. nursing rate per patient)
55. Safety evaluation
56. Patient safety reports
57. Patient safety indicators
58. Discharge and outpatient follow-up

## M3 Search Strategy

Three search terms blocks:

1. Perioperative period (Lines 1-6)
2. Patient Safety (Lines 6-20)
3. Guidelines (Lines 20-46)

Ovid MEDLINE(R) ALL / PubMed(R) <1946 to Present>

1 exp Intraoperative Complications/

2 exp Postoperative Complications/

3 exp Perioperative Care/

4 exp Perioperative Period/

5 ((peri?operative? or pre?operative? or intra?operative? or post?operative?) adj8 (adverse adj2 (effect? or reaction? or event? or "critical incident?" or incident? or complication?))).tw.

6 or/1-5

7 Patient Safety/

8 Risk Management/

9 Patient Harm/

10 Safety Management/

11 exp Malpractice/

12 exp Medical Errors/

13 patient safety.tw.

14 ((surg* or an?esth*) adj4 (safe* or error?)).tw.

15 risk management.tw.

16 (safe* adj4 (culture or intervention?)).tw.

17 (medic* adj4 (error? or mistake?)).tw.

18 malpractice?.tw.

19 human error?.tw.

20 or/7-19

21 Clinical Protocols/

22 exp Consensus/

23 exp Consensus Development Conference/

24 exp Consensus Development Conferences as Topic/

25 Critical Pathways/

26 exp Guideline/

27 Guidelines as Topic/

28 exp Practice Guideline/

29 Practice Guidelines as Topic/

30 Health Planning Guidelines/

31 Clinical Decision Rules/

32 (guideline or practice guideline or consensus development conference or consensus development conference, NIH).pt.

33 (position statement* or policy statement* or practice parameter* or best practice*).ti,ab,kf.

34 (standards or guideline or guidelines).ti,kf.

35 ((practice or treatment* or clinical) adj guideline*).ab.

36 (CPG or CPGs).ti.

37 consensus*.ab. /freq=2

38 ((critical or clinical or practice) adj2 (path or paths or pathway or pathways or protocol*)).ti,ab,kf.

39 recommendat*.ti,kf. or guideline recommendation*.ab.

40 (care adj2 (standard or path or paths or pathway or pathways or map or maps or plan or plans)).ti,ab,kf.

41 (algorithm* adj2 (screening or examination or test or tested or testing or assessment* or diagnosis or diagnoses or diagnosed or diagnosing)).ti,ab,kf.

42 (algorithm* adj2 (pharmacotherap* or chemotherap* or chemotreatment* or therap* or treatment* or intervention*)).ti,ab,kf.

43 (guideline* or standards or consensus* or recommendat*).au.

44 (guideline* or standards or consensus* or recommendat*).ca.

Embase. Elsevier

#65 #10 AND #29 AND #62 AND [embase]/lim AND [2012-2022]/py

#64 #10 AND #29 AND #62 AND [embase]/lim

#63 #10 AND #29 AND #62

#62 #30 OR #31 OR #32 OR #33 OR #34 OR #35 OR #36 OR #37 OR #38 OR #39 OR #40 OR #41 OR #42 OR #43 OR #44 OR #45 OR #46 OR #47 OR #48 OR #49 OR #50 OR #51 OR #52 OR #53 OR #54 OR #55 OR #56 OR #57 OR #58 OR #59 OR #60 OR #61

#61 guideline?:au OR standards:au OR consensus:au OR recommendat*:au

#60 'algorithm' NEAR/2 intervention?

#59 'algorithm' NEAR/2 treatment?

#58 'algorithm' NEAR/2 therap*

#57 'algorithm' NEAR/2 chemotherap*

#56 'algorithm' NEAR/2 pharmacotherap*

#55 'algorithm' NEAR/2 diagnos*

#54 'algorithm' NEAR/2 assessment?

#53 'algorithm' NEAR/2 test*

#52 'algorithm' NEAR/2 'examination'

#51 'algorithm' NEAR/2 'screening'

#50 care NEAR/2 plan?

#49 care NEAR/2 map?

#48 care NEAR/2 path*

#47 care NEAR/2 standard

#46 recommendat*:kw,ti OR 'guideline recommendation?':ab

#45 practice NEAR/2 protocol?

#44 practice NEAR/2 path*

#43 clinical NEAR/2 protocol?

#42 clinical NEAR/2 path*

#41 critical NEAR/2 protocol?

#40 critical NEAR/2 path*

#39 cpg:ti OR cpgs:ti

#38 'clinical' NEAR/1 guideline?

#37 'treatment' NEAR/1 guideline?

#36 practice NEAR/1 guideline?

#35 standard? OR guideline?:ti,kw

#34 'position statement*' OR 'policy statement*' OR 'practice parameter*' OR 'best practice*':ab,ti

#33 'clinical decision rule'/de

#32 'health care planning'/exp

#31 'practice guideline'/exp

#30 'consensus'/exp

#29 #11 OR #12 OR #13 OR #14 OR #15 OR #16 OR #17 OR #18 OR #19 OR #20 OR #21 OR #22 OR #23 OR #24 OR #25 OR #26 OR #27 OR #28

#28 'human error?':ab,ti

#27 'malpractice?':ab,ti

#26 medic* NEAR/4 mistake?

#25 medic* NEAR/4 error?

#24 safe* NEAR/4 intervention?

#23 safe* NEAR/4 culture

#22 'risk management':ab,ti

#21 an?esth* NEAR/4 error?

#20 an?esth* NEAR/4 safe*

#19 surg* NEAR/4 error?

#18 surg* NEAR/4 safe*

#17 'patient safety':ab,ti

#16 'medical error'/exp

#15 'malpractice'/de

#14 'safety culture'/de

#13 'patient harm'/de

#12 'risk management'/de

#11 'patient safety'/de

#10 #1 OR #2 OR #3 OR #4 OR #5 OR #6 OR #7 OR #8 OR #9

#9 (per?operative OR pre?operative? OR intra?operative OR post?operative) NEAR/8 'adverse incident?'

#8 (per?operative OR pre?operative? OR intra?operative OR post?operative) NEAR/8 'adverse complication?'

#7 (per?operative OR pre?operative? OR intra?operative OR post?operative) NEAR/8 'critical incident?'

#6 (per?operative OR pre?operative? OR intra?operative OR post?operative) NEAR/8 'adverse event?'

#5 (per?operative OR pre?operative? OR intra?operative OR post?operative) NEAR/8 'adverse reaction?'

#4 (per?operative OR pre?operative? OR intra?operative OR post?operative) NEAR/8 'adverse effect?'

#3 'peroperative complication'/de

#2 'perioperative period'/exp

#1 'postoperative complication'/exp

COCHRANE (WILEY)

#1 MeSH descriptor: [Intraoperative Complications] explode all trees

#2 MeSH descriptor: [Postoperative Complications] explode all trees

#3 MeSH descriptor: [Perioperative Care] explode all trees

#4 MeSH descriptor: [Perioperative Period] explode all trees

#5 (per?operative OR pre?operative? OR intra?operative OR post?operative) NEAR/8 (adverse NEAR/2 (effect? or reaction? or event? or "critical incident" or complication?)):ti,ab,kw

#6 {or #1-#5}

#7 MeSH descriptor: [Patient Safety] this term only

#8 MeSH descriptor: [Risk Management] explode all trees

#9 MeSH descriptor: [Patient Harm] this term only

#10 MeSH descriptor: [Safety Management] this term only

#11 MeSH descriptor: [Malpractice] explode all trees

#12 MeSH descriptor: [Medical Errors] explode all trees

#13 patient safety:ti,ab,kw

#14 ((surg* or an?esth*) NEAR/4 (safe* or error?)):ti,ab,kw

#15 risk management:ti,ab,kw

#16 (safe* NEAR/4 (culture or intervention?)):ti,ab,kw

#17 (medic* NEAR/4 (error? or mistake?)):ti,ab,kw

#18 malpractice?:ti,ab,kw

#19 human error?:ti,ab,kw

#20 {or #7-#19}

#21 MeSH descriptor: [Clinical Protocols] this term only

#22 MeSH descriptor: [Consensus] explode all trees

#23 MeSH descriptor: [Critical Pathways] explode all trees

#24 MeSH descriptor: [Guidelines as Topic] explode all trees

#25 MeSH descriptor: [Consensus Development Conferences as Topic] explode all trees

#26 MeSH descriptor: [Practice Guidelines as Topic] explode all trees

#27 MeSH descriptor: [Health Planning Guidelines] explode all trees

#28 MeSH descriptor: [Clinical Decision Rules] explode all trees

#29 (guideline or practice guideline or consensus development conference or consensus development conference, NIH):pt

#30 (position statement* or policy statement* or practice parameter* or best practice*):ti,ab,kw

#31 (standards or guideline or guidelines):ti,kw

#32 ((practice or treatment? or clinical) NEXT guideline?):ab

#33 (CPG or CPGs):ti

#34 (consens*):ab

#35 (critical or clinical or practice) NEAR/2 (path* or protocol?):ti,ab,kw

#36 recommendat*:ti,kw or guideline recommendation?:ab

#37 care NEAR/2 (standard or path* or map? or plan?):ti,ab,kw

#38 (algorithm* NEAR/2 (pharmacotherap* or chemotherap* or chemotreatment? or therap* or treatment? or intervention?)):ti,ab,kw

#39 (guideline? or standards or consens? or recommendat*):au

#40 {or #21-#39}

#41 #6 and #20 and #40 with Publication Year from 2012 to 2022, in Trials

Virtual Health Library (VHL) Regional Portal

Title, Abstract, Subject: (intraoperative OR postoperative OR perioperative OR preoperative) AND "patient safety" AND ("clinical protocols" OR consensus OR guideline* OR "practice guideline" OR standards OR recommendation*) AND ( db:("LILACS" OR "IBECS" OR "BDENF" OR "BINACIS" OR "BIGG" OR "SES-SP" OR "WHOLIS")) AND (year_cluster:[2012 TO 2022])

TRIPDATABASE

(intraoperative OR postoperative OR perioperative OR preoperative) AND "patient safety" AND ("clinical protocols" OR consensus OR guideline* OR "practice guideline" OR standards OR recommendation*) from_date:2014

NOTE: in this database 2014 is the first year available for filtering

## M4 Data extraction forms and variables description

### Table M4.1 Data extraction database for Guidelines

| **Var ID** | **Variable** | **Variable short name** | **Explanation** | **Type of variable** | **Coding or dropdown items** |
| --- | --- | --- | --- | --- | --- |
| **1** | **Guide_n** | Guideline number | Autonumeric | number | 1, 2, 3... |
| **2** | **Guideline ID** | Guideline ID | First author's surname and year to identify the document. | text | e.g. Anne, 2021 |
| **3** | **Year of publication** | Year of publication | Publication year of the guideline | number | e.g. 2022 |
| **4** | **Guideline Title** | Guideline Title | Guideline title | text | N/A |
| **5** | **Type of organization** | Type of organization | Refers to the organization profile. | categorical | Public institution / Private organism / Scientific community / Other |
| **6** | **Type of publication** | Type of publication | Type of guideline publication based on operational definitions | categorical | CPG/ Position statements/ Expert consensus/ Others (see below) |
| **7** | **Number of Recommendations** | Number of recommendations | Number of total recommendations in the guideline | number | e.g. 34 |
| **8** | **Income level** | Income level of the publishing country or region | Income level of the geographical area from the recommendations were developed using World Bank’s data | categorical | Income level from a country or region list (see below) |
| **9** | **Original Language** | Original Language | Language employed to write the document | categorical | Language list (see below) |
| **10** | **Scope** | Scope | Target scope of the recommendations | categorical | International/ National / Regional (see below) |
| **11** | **New guideline or updated** | New guideline or updated | It is a newly created guideline or an updated version of a previous one. | dicotomous | New / Updated |
| **12** | **Method to formulate recommendations** | Method to formulate recommendations | Method employed to formulate level of evidence and/or strength of the recommendations | categorical | Name of the grading system and year if more than one version is known (e.g. GRADE / Oxford CEBM (2009) / Oxford CEBM (2011) / … ) |

Definition of specific variables of the Guidelines Extraction Form

Type of organization

- Public institution: Any entity established or controlled by the government (central, state or local);
- Private organism: Independent entity owned by a non-State entity;
- Scientific community: A group organized explicitly for the purpose of advancing science, whether they are represented by an organism or publishing in collaborative manner;
- Other: Any other not considered in the previous categories.

Type of publication

Adapted from Joshi et al. 2019^1^:

- CPG (Clinical practice guideline): Systematically developed documents created with a validated methodology, which includes identifying the literature on specific clinical question(s), characterized by explicit methods of searching, selection, and grading the available evidence;
- Position statements: Comprehensive document that elucidates, justifies and recommends a particular approach to a clinical problem;
- Expert consensus: Recommendations developed based on a collective opinion or consensus of the convened expert panel;
- Other: Document of Recommendations on a medical condition developed without following a certain procedure.

1. Joshi GP, Benzon HT, Gan TJ, Vetter TR. Consistent Definitions of Clinical Practice Guidelines, Consensus Statements, Position Statements, and Practice Alerts. Anesthesia & Analgesia 129(6):p 1767-1770, Dec 2019. DOI: 10.1213/ANE.0000000000004236

Income level of the publishing country/region

Income level classified using world bank 2023 data.^2^

1. World Bank. World Bank: country and lending groups. World Bank Data Website. 2023; <https://datahelpdesk.worldbank.org/knowledgebase/articles/906519>. Accessed 24 Nov 2023

Original Language

ISO 639 Language Codes were used.^3^

1. International Organization for Standardization. ISO 639:2023. Code for individual languages and language groups. International Organization for Standardization website. 2023; <https://www.iso.org/standard/74575.html>. Accessed 24 Nov 2023

Scope

- International: Aimed at supranational level or it is a between-nations collaboration;
- National: Aimed at national level;
- Regional: Aimed at subnational level (e.g. a particular region, district, area, local context or part of a country).

### Table M4.2 Data extraction form for Recommendations

| **Var ID** | **Variable** | **Variable short name** | **Explanation** | **Type of variable** | **Coding or Dropdown items** |
| --- | --- | --- | --- | --- | --- |
| **1** | **R_n** | Recommendation number | Autonumeric | number | 1, 2, 3... |
| **2** | **Guideline ID** | Guideline ID | First author's surname and year to identify the document. | text | e.g. Anne, 2021 |
| **3** | **Full-length recommendation** | Full-length recommendation | Verbatim quote of the full-length recommendation from the original document. | text | e.g. It is recommended that mechanical bowel preparation not be used routinely to prevent SSI. |
| **4** | **Perioperative period** | Perioperative period | Perioperative period in which recommendation is mainly focused on. | categorical | Preadmission / Preoperative / Intraoperative / Postoperative / Post-discharge / Mixed |
| **5** | **Clinical setting** | Clinical setting | Applicability of the recommendation to a clinical setting in terms of a hospital admission or an outpatient context (i.e. ambulatory or day-surgery patients) | categorical | Outpatient / Inpatient / Both |
| **6** | **Surgical patient safety area** | Surgical patient safety area | Predefined surgical patient safety area in which recommendation could be classified. | categorical | (See above in section M2 of this document) |
| **7** | **Surgical patient safety subarea** | Surgical patient safety subarea | Predefined surgical patient safety subarea in which recommendation could be classified. | categorical | (See above in section M2 of this document) |
| **8** | **Level of evidence** | Level of evidence | Level of evidence as referred in the guideline (i.e. verbatim quote). If not mentioned, "Not reported" is provided. | text | eg. Low quality |
| **9** | **Strength of recommendation** | Strength of recommendation | Strength of recommendation as it is described in the guideline (i.e. verbatim quote). If not mentioned, "Not reported" is provided. | text | eg. Strong |

## M5 Levels of Evidence algorithm. Methodological explanation

As it is mentioned in its handbook^1^, the GRADE approach to grading the quality of evidence is initially established based on the study design (trials or observational studies) and then other reasons are considered to rate down or up the quality of evidence.

We based our approach on the first publications from the GRADE group that alluded to three grades of evidence^2,3^:

- **Randomized trials**, that could be initially interpreted as **high**.
- **Observational studies**, that could be interpreted as **low.**
- And **any other evidence**, that could be interpreted as **very low.**

We then included other study types not previously considered as well as **systematic reviews of randomized controlled trials**, modulating the **high** level of evidence in the presence of multiple high-quality randomized controlled trials and downgrading it to moderate if it is represented by single or not appraised trials, as shown in Figure 1.

**Table M5.1 Study type proxies defined in our algorithm.**

| **GRADE (letters)** | **GRADE (qualifier)** | **Study type (proxy)** |
| --- | --- | --- |
| A | High | Systematic reviews of randomized controlled trials, with or without meta-analysis, or Multiple high-quality randomized controlled trials. |
| B | Moderate | One or multiple randomized controlled trials without quality appraisal considerations, Systematic reviews of Observational Studies. |
| C | Low | Quasi-experimental studies, Observational Studies. |
| D | Very Low | Case studies, Expert Opinion, Legislation, Regulations and Standards. |

Finally, two additional remarks were considered when establishing a translation across scales. Firstly, several classifiers of a grading system were allowed to be at the same GRADE level, but none can be repeated at more than one GRADE level (the translation would not allow for mutually exclusive levels of evidence if done otherwise).

And secondly, when doubting between two levels of GRADE equivalence, a downgrading was conducted taking the most conservative equivalence (i.e. the lowest among those considered) in order to avoid misclassifying upwards, primarily in the absence of a proper explanation ensuring that a thorough quality appraisal was performed or only good quality studies were considered in a particular classifier of a grading system.

All equivalences are presented in the table below.

**Table M5.2 Equivalences algorithm**

| **GRADE *in* *letters*** | A | B | C | D |
| --- | --- | --- | --- | --- |
| **GRADE *in* *qualifier*** | High | Moderate | Low | Very Low |
| **AACE (2017)**^4^ | 1 | - | 2 | 3, 4 |
| **AAOS (2019)**^5–7^ | Strong | - | Moderate | Low Strength or Conflicting Evidence; No Evidence |
| **ACC/AHA (2014)**^8–11^ | Level A | Level B | - | Level C |
| **ACCF/AHA (2019)**^12–16^ | Level A | Level B-R | Level B-NR | Level C-LD, Level C-EO |
| **ACP (2010)**^17,18^ | High | Moderate | Low | - |
| **AORN (2022)**^19^ | IA, IB | IIA, IIB | IIIA, IIIB, IC, IIC | IV, V, IIIC |
| **APSIC (2019)**^20^ | - | I | II | III |
| **ASA (2012)**^21^ | A1 | A2, A3 | B1 | B2, B3 |
| **ASA (2016)**^22–25^ | A1 | A2, A3 | B1 | B2, B3, B4 |
| **ASHP (1995)**^26^ | I | II | III, IV, V | VI, VII |
| **ASRA (2018)**^27^ | A | B | - | C |
| **Benavides et al. (2015)**^28^ | A1 | A2, A3 | B1, B2 | B3, B4, Insufficient |
| **ESC (2010)**^29–36^ | A | B | - | C |
| **HPSC (2005)**^37^ | A | B | C | D |
| **IDSA (2001)**^38^ | - | I | II | III |
| **Malcolm et al. (2018)**^39^ | 1A | 1B, 1 (diagnosis or prognosis) | 2, 3 | 4 |
| **NHRMC (2009) *body of evidence matrix & levels***^40^ | A, I | B, II | C, III-1, III-2, III-3 | D, IV |
| **Oxford CEBM (2009)**^41–43^ | 1a | 1b, 1c, 2a | 2b, 2c, 3a | 3b, 4, 5 |
| **Oxford CEBM (2011)**^44–46^ | 1 | 2 | 3 | 4, 5 |
| **RCoA (2016)**^47–58^ | A | B | - | C, M, GPP |
| **Rubino et al. (2016)**^59^ | IA | IB | IIA, IIB, III | IV |
| **Shekelle (1999)**^60–66^ | Ia | Ib | IIa, IIb, III | IV |
| **SIGN (2012)**^60,65–67^ | 1++, 1+ | 1-, 2++ | 2+, 2- | 3, 4 |
| **SIGN (2019)**^68,69^ | 1++, 1+ | 1-, 2++ | 2+, 2- | 3, 4 |
| **SORT *letters & numbers***^70^ | A, 1 | - | B, 2 | C, 3 |
| **Adapted from OXFORD**^71^ | A, 1, Ia, Ib | B, 2, IIa | C, 3, 4, IIb, III | D, 5, Expert consensus |
| **Adapted from Sackett**^65^ | 1 | 2 | 3 | 4, 5 |
| **Adapted from SIGN**^72,73^ | Ia, 1 | Ib, 2 | IIa, IIb, III | IV, 3, 4 |
| **Adapted from USPSTF**^74^ | - | I, II-1 | II-2, II-3 | III |

# Supplementary Appendixes

## A1 Prisma 2020 Checklist

| **Section and Topic** | **Item #** | **Checklist item** | **Location where item is reported** |
| --- | --- | --- | --- |
| **TITLE** | | |  |
| Title | 1 | Identify the report as a systematic review. | Page 1 |
| **ABSTRACT** | | |  |
| Abstract | 2 | See the PRISMA 2020 for Abstracts checklist. | Page 1 |
| **INTRODUCTION** | | |  |
| Rationale | 3 | Describe the rationale for the review in the context of existing knowledge. | Page 3 |
| Objectives | 4 | Provide an explicit statement of the objective(s) or question(s) the review addresses. | Page 3 |
| **METHODS** | | |  |
| Eligibility criteria | 5 | Specify the inclusion and exclusion criteria for the review and how studies were grouped for the syntheses. | Page 3 (“eligibility criteria” section) and SDC 1 |
| Information sources | 6 | Specify all databases, registers, websites, organisations, reference lists and other sources searched or consulted to identify studies. Specify the date when each source was last searched or consulted. | Page 4 |
| Search strategy | 7 | Present the full search strategies for all databases, registers and websites, including any filters and limits used. | SDC 1 (Page 4 for clarification about limits) |
| Selection process | 8 | Specify the methods used to decide whether a study met the inclusion criteria of the review, including how many reviewers screened each record and each report retrieved, whether they worked independently, and if applicable, details of automation tools used in the process. | Page 4 (“Screening”) |
| Data collection process | 9 | Specify the methods used to collect data from reports, including how many reviewers collected data from each report, whether they worked independently, any processes for obtaining or confirming data from study investigators, and if applicable, details of automation tools used in the process. | Pages 4-5 (“Data extraction”) |
| Data items | 10a | List and define all outcomes for which data were sought. Specify whether all results that were compatible with each outcome domain in each study were sought (e.g. for all measures, time points, analyses), and if not, the methods used to decide which results to collect. | SDC 1 and text in pages 4-5 |
|  | 10b | List and define all other variables for which data were sought (e.g. participant and intervention characteristics, funding sources). Describe any assumptions made about any missing or unclear information. | SDC 1 and text in pages 4-5 |
| Study risk of bias assessment | 11 | Specify the methods used to assess risk of bias in the included studies, including details of the tool(s) used, how many reviewers assessed each study and whether they worked independently, and if applicable, details of automation tools used in the process. | Pages 5 (“Quality assessment”) |
| Effect measures | 12 | Specify for each outcome the effect measure(s) (e.g. risk ratio, mean difference) used in the synthesis or presentation of results. | N.A. |
| Synthesis methods | 13a | Describe the processes used to decide which studies were eligible for each synthesis (e.g. tabulating the study intervention characteristics and comparing against the planned groups for each synthesis (item #5)). | Page 6 (“Statistical analysis”) |
|  | 13b | Describe any methods required to prepare the data for presentation or synthesis, such as handling of missing summary statistics, or data conversions. | Pages 5-6 (“Qualitative analysis”, “Quality assessment” and “Statistical analysis” sections) |
|  | 13c | Describe any methods used to tabulate or visually display results of individual studies and syntheses. | Page 6 (“Statistical analysis”) |
|  | 13d | Describe any methods used to synthesize results and provide a rationale for the choice(s). If meta-analysis was performed, describe the model(s), method(s) to identify the presence and extent of statistical heterogeneity, and software package(s) used. | Pages 5-6 (“Qualitative analysis”, and “Statistical analysis” sections) |
|  | 13e | Describe any methods used to explore possible causes of heterogeneity among study results (e.g. subgroup analysis, meta-regression). | Page 6 (“Statistical analysis”) |
|  | 13f | Describe any sensitivity analyses conducted to assess robustness of the synthesized results. | N.A. |
| Reporting bias assessment | 14 | Describe any methods used to assess risk of bias due to missing results in a synthesis (arising from reporting biases). | N.A. |
| Certainty assessment | 15 | Describe any methods used to assess certainty (or confidence) in the body of evidence for an outcome. | N.A. |
| **RESULTS** | | |  |
| Study selection | 16a | Describe the results of the search and selection process, from the number of records identified in the search to the number of studies included in the review, ideally using a flow diagram. | Figure 1 and text in page 6. |
|  | 16b | Cite studies that might appear to meet the inclusion criteria, but which were excluded, and explain why they were excluded. | Figure 1 |
| Study characteristics | 17 | Cite each included study and present its characteristics. | SDC 3, Table 1 and page 6 |
| Risk of bias in studies | 18 | Present assessments of risk of bias for each included study. | Table 1, Table 3, and SDC 3 |
| Results of individual studies | 19 | For all outcomes, present, for each study: (a) summary statistics for each group (where appropriate) and (b) an effect estimate and its precision (e.g. confidence/credible interval), ideally using structured tables or plots. | Tables 1-S4 (N.A. for effect estimates) |
| Results of syntheses | 20a | For each synthesis, briefly summarise the characteristics and risk of bias among contributing studies. | Table 3 |
|  | 20b | Present results of all statistical syntheses conducted. If meta-analysis was done, present for each the summary estimate and its precision (e.g. confidence/credible interval) and measures of statistical heterogeneity. If comparing groups, describe the direction of the effect. | Tables 3-S5 (N.A. for meta-analysis) |
|  | 20c | Present results of all investigations of possible causes of heterogeneity among study results. | Table 1 and 2. |
|  | 20d | Present results of all sensitivity analyses conducted to assess the robustness of the synthesized results. | N.A. |
| Reporting biases | 21 | Present assessments of risk of bias due to missing results (arising from reporting biases) for each synthesis assessed. | N.A. |
| Certainty of evidence | 22 | Present assessments of certainty (or confidence) in the body of evidence for each outcome assessed. | N.A. However, level of evidence is analyzed for recommendations. See Tables 2 and S4, as well as SDC 2, 4 and 6. |
| **DISCUSSION** | | |  |
| Discussion | 23a | Provide a general interpretation of the results in the context of other evidence. | Fourth paragraph of “Discussion” (Page 9) |
|  | 23b | Discuss any limitations of the evidence included in the review. | Page 10 |
|  | 23c | Discuss any limitations of the review processes used. | Page 10 |
|  | 23d | Discuss implications of the results for practice, policy, and future research. | Pages 9-10 (sixth and last paragraph of “Discussion”) |
| **OTHER INFORMATION** | | |  |
| Registration and protocol | 24a | Provide registration information for the review, including register name and registration number, or state that the review was not registered. | Page 2 (below “Abstract”) and page 3 (first paragraph in “Materials and methods”) |
|  | 24b | Indicate where the review protocol can be accessed, or state that a protocol was not prepared. | Page 2 (below “Abstract”) and page 3 (first paragraph in “Materials and methods”) |
|  | 24c | Describe and explain any amendments to information provided at registration or in the protocol. | N.A. |
| Support | 25 | Describe sources of financial or non-financial support for the review, and the role of the funders or sponsors in the review. | Title page (“Acknowledgments”) |
| Competing interests | 26 | Declare any competing interests of review authors. | Title page (“Conflict of interests”) |
| Availability of data, code and other materials | 27 | Report which of the following are publicly available and where they can be found: template data collection forms; data extracted from included studies; data used for all analyses; analytic code; any other materials used in the review. | Supplementary material |

*From:*  Page MJ, McKenzie JE, Bossuyt PM, Boutron I, Hoffmann TC, Mulrow CD, et al. The PRISMA 2020 statement: an updated guideline for reporting systematic reviews. BMJ 2021;372:n71. doi: 10.1136/bmj.n71

For more information, visit: <http://www.prisma-statement.org/>

# Supplementary Figures and Tables

**Table S1. High-quality Clinical Practice Guidelines containing tools or materials for supporting their implementation.**

| **CPG_ID** | **Reference** | **Structured literature review** | **Method for grading recommendations** | **External review** | **AGREE-II Rigour of development** | **Tools and materials for supporting implementation** | **Tools and materials (originals)** | **Tools and materials (cited or adapted)** | **Indicators for monitoring and evaluation** |
| --- | --- | --- | --- | --- | --- | --- | --- | --- | --- |
| Anne, 2021 | Anne S, Mims JW, Tunkel DE, Rosenfeld RM, Boisoneau DS, Brenner MJ, et al. Clinical Practice Guideline: Opioid Prescribing for Analgesia After Common Otolaryngology Operations. Otolaryngol Neck Surg [Internet]. 2021;164:S1–42. Available from: https://onlinelibrary.wiley.com/doi/10.1177/0194599821996297 | Yes | Adapted from Oxford CEBM | Yes | 84.4 | Yes | Defining Mild, Moderate, and Severe Pain for the Patient; Secure storage and disposal of opioids (infographic); Opioid guideline key action statements (algorithm). | SOS: Risk Score; DSM-5 Diagnostic Criteria for Opioid Use Disorder; Patient education materials on postoperative pain control and opioid use. NSAIDs, nonsteroidal anti-inflammatory drugs. | No |
| AORN, 2022a | AORN. Guidelines for perioperative practices. 2022 edition. Denver: Association of periOperative Registered Nurses; 2022. 1–1274 p. | Yes | AORN (2022) | Yes | 79.2 | Yes | (Mostly evaluation tools and decisional tools) |  | No |
| Apfelbaum, 2021 | Apfelbaum JL, Hagberg CA, Connis RT, Abdelmalak BB, Agarkar M, Dutton RP, et al. 2022 American Society of Anesthesiologists Practice Guidelines for Management of the Difficult Airway. Anesthesiology [Internet]. 2022;136:31–81. Available from: https://pubs.asahq.org/anesthesiology/article/136/1/31/117915/2022-American-Society-of-Anesthesiologists | Yes | ASA (2016) | Yes | 71.9 | Yes | Difficult airway algorithm; Difficult airway infographic |  | No |
| Brunt, 2020 | Brunt LM, Deziel DJ, Telem DA, Strasberg SM, Aggarwal R, Asbun H, et al. Safe Cholecystectomy Multi-society Practice Guideline and State of the Art Consensus Conference on Prevention of Bile Duct Injury During Cholecystectomy. Ann Surg [Internet]. 2020;272:3–23. Available from: https://journals.lww.com/10.1097/SLA.0000000000003791 | Yes | GRADE | Yes | 91.7 | Yes | (Several online didactic modules) |  | No |
| Devlin, 2018 | Devlin JW, Skrobik Y, Gélinas C, Needham DM, Slooter AJC, Pandharipande PP, et al. Clinical Practice Guidelines for the Prevention and Management of Pain, Agitation/Sedation, Delirium, Immobility, and Sleep Disruption in Adult Patients in the ICU. Crit Care Med. 2018;46:E825–73. | Yes | GRADE | No | 81.3 | Yes | As a separated publication. (Balas MC, Weinhouse GL, Denehy L, et al: Interpreting and implementing the 2018 Pain, Agitation/Sedation, Delirium, Immobility, and Sleep Disruption Clinical Practice Guideline. Crit Care Med 2018; 46:1464–1470) |  | No |
| IMSS, 2013a | IMSS. Prevención y Manejo de las Complicaciones Postoperatorias en Cirugía no Cardiaca en el Adulto Mayor [Internet]. Coordinación de Unidades Médicas de Alta Especialidad, editor. Mexico: Instituto Mexicano del Seguro Social; 2013 [cited 2022 Sep 21]. Available from: http://www.imss.gob.mx/profesionales/guiasclinicas/Pages/guias.aspx | Yes | Several scales used | Yes | 70.8 | Yes | General Algorithm for Postoperative Complications; Specific treatment of postoperative pulmonary complications; Approach to postoperative delirium; Assessment for the use of the route of administration of nutritional support in the hospitalized elderly. | Nomogram for the administration of Unfractionated Heparin; ASA (American Society of Anesthesiologists) preoperative risk classification; Differential diagnosis of postoperative delirium and POCD; Folstein mini mental exam; Confusion Assessment Method. | No |
| IMSS, 2013b | IMSS. Intervenciones Preventivas Para la Seguridad en el Paciente Quirúrgico [Internet]. Coordinación de Unidades Médicas de Alta Especialidad, editor. Mexico: Instituto Mexicano del Seguro Social; 2013 [cited 2022 Sep 21]. Available from: http://www.imss.gob.mx/profesionales/guiasclinicas/Pages/guias.aspx | Yes | Shekelle (1999) and GRADE | Yes | 74.0 | Yes |  | Surgical Safety Checklist (WHO) | No |
| Korytkowski, 2022 | Korytkowski MT, Muniyappa R, Antinori-Lent K, Donihi AC, Drincic AT, Hirsch IB, et al. Management of Hyperglycemia in Hospitalized Adult Patients in Non-Critical Care Settings: An Endocrine Society Clinical Practice Guideline. J Clin Endocrinol Metab [Internet]. 2022;107:2101–28. Available from: https://academic.oup.com/jcem/article/107/8/2101/6605637 | Yes | GRADE | Yes | 84.4 | Yes | Methodology for converting continuous subcutaneous insulin infusion to scheduled basal bolus insulin. | Resources required for safe implementation of continuous glucose monitoring in the noncritical care hospital setting Engagement. | No |
| Nast, 2021 | Nast A, Häfner HM, Kolk A, Koscielny J, Kunte C, Löser C, et al. S3 guideline: Management of anticoagulants and antiplatelet agents in cutaneous surgery. JDDG - J Ger Soc Dermatology [Internet]. 2021 [cited 2023 Aug 1];19:1531–46. Available from: https://register.awmf.org/de/leitlinien/detail/013-085 | Yes | GRADE | Yes | 74.0 | Yes | Algorithms (ATB) | Decision/cognitive aid for ATB in skin surgery | No |
| WHO, 2018 | WHO. Global guidelines for the prevention of surgical site infection. [Internet]. Second edition. Geneva: World Health Organization; 2018 [cited 2022 Dec 28]. 184 p. Available from: https://apps.who.int/iris/rest/bitstreams/1168437/retrieve | Yes | GRADE | Yes | 80.2 | Yes | (Web appendices; implementation strategy document and tool package) |  | No |
| Department of Health, 2020 | Department of Health. Nutrition screening and use of oral nutrition support for adults in the acute care setting [Internet]. Dublin; 2020 [cited 2022 Dec 28]. (22). Report No.: 22. Available from: http://health.gov.ie/en/collection/c9fa9a-national-clinical-guidelines/ | Yes | SIGN and GRADE | Yes | 87.5 | Yes | Implementation plan and logic model | Criteria for diagnosing malnutrition; HSE Food Nutrition and Hydration Policy algorithm; Supporting tools (compilation) | Yes |
| RNAO, 2019 | RNAO. Supporting Adults Who Anticipate or Live with an Ostomy [Internet]. 2nd ed. Toronto: Registered Nurses’ Association of Ontario; 2019. Available from: www.RNAO.ca/bpg | Yes | GRADE | Yes | 100.0 | Yes | Sample Ostomy Teaching Record; Enhancing Your Recovery after Ostomy Surgery: Your Personal Checklist | Tools and resources (websites) | Yes |
| Boselli, 2020 | Boselli E, Hopkins P, Lamperti M, Estèbe JP, Fuzier R, Biasucci DG, et al. European Society of Anaesthesiology and Intensive Care Guidelines on peri-operative use of ultrasound for regional anaesthesia (PERSEUS regional anesthesia). Eur J Anaesthesiol [Internet]. 2020;38:219–50. Available from: https://journals.lww.com/10.1097/EJA.0000000000001383 | Yes | Adapted from GRADE | Yes | 82.3 | No |  |  | Yes |

**Table S2. Distribution across perioperative period and level of evidence of the strong recommendations from high-quality clinical practice guidelines (“rigour of development” > 70%) (n=562)**

|  | | | | | | | | | | | | | |
| --- | --- | --- | --- | --- | --- | --- | --- | --- | --- | --- | --- | --- | --- |
|  | | **Perioperative period (% by patient safety area)** | | | | | | **Normalized level of evidence (% by patient safety area)** | | | | |  |
|  | **Pre-admission** | | **Pre-operative** | **Intraoperative** | **Post-operative** | **Post-discharge** | **Mixed** | **A** | **B** | **C** | **D** | **Not reported** | **Total (% over grand total)** |
| **Diagnosis and referral** | - | | - | - | - | - | - | - | - | - | - | - | 0 (0) |
| **Preoperative evaluation and planning** | 5 (20) | | 10 (40) | 3 (12) | 2 (8) | - | 5 (20) | 3 (12) | 3 (12) | 6 (24) | 6 (24) | 7 (28) | 25 (4.4) |
| **Patient information and communication** | 4 (30.8) | | - | - | 1 (7.7) | 6 (46.2) | 2 (15.4) | 1 (7.7) | 2 (15.4) | 9 (69.2) | - | 1 (7.7) | 13 (2.3) |
| **Healthcare provider communication and handovers** | - | | 1 (2.6) | 20 (52.6) | 1 (2.6) | - | 16 (42.1) | 2 (5.3) | 6 (15.8) | 9 (23.7) | 15 (39.5) | 6 (15.8) | 38 (6.8) |
| **Monitoring and registries** | - | | - | 4 (40) | - | - | 6 (60) | 1 (10) | - | 3 (30) | 4 (40) | 2 (20) | 10 (1.8) |
| **Patient support and complication prevention** | 6 (2.8) | | 12 (5.6) | 154 (72) | 17 (7.9) | - | 25 (11.7) | 20 (9.3) | 34 (15.9) | 74 (34.6) | 72 (33.6) | 14 (6.5) | 214 (38.1) |
| **Standard surgical and anaesthetic procedures** | - | | 4 (10.5) | 20 (52.6) | 1 (2.6) | 2 (5.3) | 11 (28.9) | 7 (18.4) | 9 (23.7) | 11 (28.9) | 8 (21.1) | 3 (7.9) | 38 (6.8) |
| **Safe medication use** | - | | - | 2 (5.3) | - | - | 36 (94.7) | 3 (7.9) | 1 (2.6) | 15 (39.5) | 15 (39.5) | 4 (10.5) | 38 (6.8) |
| **Safe blood derivates management** | - | | - | - | - | - | 1 (100) | - | - | 1 (100) | - | - | 1 (0.2) |
| **Health care infection prevention** | 6 (5.6) | | 18 (16.8) | 50 (46.7) | 2 (1.9) | - | 31 (29) | 36 (33.6) | 14 (13.1) | 19 (17.8) | 25 (23.4) | 13 (12.1) | 107 (19) |
| **Safe Equipment and set up** | - | | 3 (10.7) | 3 (10.7) | 1 (3.6) | - | 21 (75) | - | 1 (3.6) | 3 (10.7) | 21 (75) | 3 (10.7) | 28 (5) |
| **Safety structures** | - | | - | 4 (15.4) | - | - | 22 (84.6) | 3 (11.5) | 2 (7.7) | 10 (38.5) | 9 (34.6) | 2 (7.7) | 26 (4.6) |
| **Human resources** | - | | - | 6 (28.6) | 1 (4.8) | - | 14 (66.7) | 2 (9.5) | 2 (9.5) | 9 (42.9) | 6 (28.6) | 2 (9.5) | 21 (3.7) |
| **Safety evaluation** | - | | - | 1 (50) | - | - | 1 (50) | - | - | 1 (50) | - | 1 (50) | 2 (0.4) |
| **Discharge and outpatient follow-up** | - | | - | - | - | 1 (100) | - | - | - | - | - | 1 (100) | 1 (0.2) |
| Values are absolute frequency and percentages in parentheses unless indicated otherwise. | | | | | | | | | | | | | |

# References

### References M5

1. The GRADE Working Group. GRADE handbook for grading quality of evidence and strength of recommendations. [Internet]. Oct 2013. Schünemann H, Brożek J, Guyatt G, Oxman A, editors. 2013. Available from: guidelinedevelopment.org/handbook

2. Atkins D, Best D, Briss PA, Eccles M, Falck-Ytter Y, Flottorp S, et al. Grading quality of evidence and strength of recommendations. BMJ [Internet]. 2004;328:1490. Available from: https://www.bmj.com/lookup/doi/10.1136/bmj.328.7454.1490

3. Guyatt GH, Oxman AD, Vist GE, Kunz R, Falck-Ytter Y, Alonso-Coello P, et al. GRADE: an emerging consensus on rating quality of evidence and strength of recommendations. BMJ [Internet]. 2008;336:924–6. Available from: http://www.ncbi.nlm.nih.gov/pubmed/18436948

4. Mechanick JI, Apovian C, Brethauer S, Garvey WT, Joffe AM, Kim J, et al. Clinical practice guidelines for the perioperative nutrition, metabolic, and nonsurgical support of patients undergoing bariatric procedures – 2019 update: cosponsored by American Association of Clinical Endocrinologists/American College of Endocrinology,. Surgery for Obesity and Related Diseases [Internet]. 2020;16:175–247. Available from: https://linkinghub.elsevier.com/retrieve/pii/S1550728919310792

5. AAOS. Management of Hip Fractures in Older Adults Evidence-Based Clinical Practice Guideline [Internet]. Rosemont; 2021 Dec. Available from: https://www.aaos.org/hipfxcpg

6. AAOS. Evaluation of Psychosocial Factors Influencing Recovery from Adult Orthopaedic Trauma Evidence-Based Clinical Practice Guideline [Internet]. 2019. Available from: www.orthoguidelines.org

7. AAOS. Management of Osteoarthritis of the Hip Evidence-Based Clinical Practice Guideline [Internet]. 2017. Available from: www.orthoguidelines.org

8. AATS Surgical Treatment of Infective Endocarditis Consensus Guidelines Writing Committee Chairs, Pettersson GB, Coselli JS, Writing Committee, Pettersson GB, Coselli JS, et al. 2016 The American Association for Thoracic Surgery (AATS) consensus guidelines: Surgical treatment of infective endocarditis: Executive summary. J Thorac Cardiovasc Surg [Internet]. 2017;153:1241-1258.e29. Available from: http://www.ncbi.nlm.nih.gov/pubmed/28365016

9. Amsterdam EA, Wenger NK, Brindis RG, Casey DE, Ganiats TG, Holmes DR, et al. 2014 AHA/ACC Guideline for the Management of Patients With Non–ST-Elevation Acute Coronary Syndromes. J Am Coll Cardiol [Internet]. 2014;64:e139–228. Available from: https://linkinghub.elsevier.com/retrieve/pii/S0735109714062792

10. Fleisher LA, Fleischmann KE, Auerbach AD, Barnason SA, Beckman JA, Bozkurt B, et al. 2014 ACC/AHA Guideline on Perioperative Cardiovascular Evaluation and Management of Patients Undergoing Noncardiac Surgery. J Am Coll Cardiol [Internet]. 2014;64:e77–137. Available from: https://linkinghub.elsevier.com/retrieve/pii/S0735109714055363

11. Shen KR, Bribriesco A, Crabtree T, Denlinger C, Eby J, Eiken P, et al. The American Association for Thoracic Surgery consensus guidelines for the management of empyema. J Thorac Cardiovasc Surg [Internet]. 2017;153:e129–46. Available from: https://linkinghub.elsevier.com/retrieve/pii/S0022522317301526

12. Lele A V, Hoefnagel AL, Schloemerkemper N, Wyler DA, Chaikittisilpa N, Vavilala MS, et al. Perioperative Management of Adult Patients With External Ventricular and Lumbar Drains: Guidelines From the Society for Neuroscience in Anesthesiology and Critical Care. J Neurosurg Anesthesiol [Internet]. 2017;29:191–210. Available from: http://www.ncbi.nlm.nih.gov/pubmed/28169966

13. Otto CM, Nishimura RA, Bonow RO, Carabello BA, Erwin JP, Gentile F, et al. 2020 ACC/AHA Guideline for the Management of Patients With Valvular Heart Disease. J Am Coll Cardiol [Internet]. 2021;77:e25–197. Available from: https://linkinghub.elsevier.com/retrieve/pii/S0735109720377962

14. Tibi P, McClure RS, Huang J, Baker RA, Fitzgerald D, Mazer CD, et al. STS/SCA/AmSECT/SABM Update to the Clinical Practice Guidelines on Patient Blood Management. Ann Thorac Surg [Internet]. 2021;112:981–1004. Available from: https://linkinghub.elsevier.com/retrieve/pii/S0003497521005567

15. Chrimes N, Higgs A, Hagberg CA, Baker PA, Cooper RM, Greif R, et al. Preventing unrecognised oesophageal intubation: a consensus guideline from the Project for Universal Management of Airways and international airway societies*. Anaesthesia [Internet]. 2022 [cited 2022 Sep 22];77:1395–415. Available from: https://onlinelibrary.wiley.com/doi/10.1111/anae.15817

16. Engelman DT, Ben Ali W, Williams JB, Perrault LP, Reddy VS, Arora RC, et al. Guidelines for Perioperative Care in Cardiac Surgery. JAMA Surg [Internet]. 2019 [cited 2022 Sep 22];154:755. Available from: https://jamanetwork.com/journals/jamasurgery/fullarticle/2732511

17. American Geriatrics Society Expert Panel on Postoperative Delirium in Older Adults. American Geriatrics Society Abstracted Clinical Practice Guideline for Postoperative Delirium in Older Adults. J Am Geriatr Soc [Internet]. 2015;63:142–50. Available from: https://onlinelibrary.wiley.com/doi/10.1111/jgs.13281

18. Patel KN, Yip L, Lubitz CC, Grubbs EG, Miller BS, Shen W, et al. Executive Summary of the American Association of Endocrine Surgeons Guidelines for the Definitive Surgical Management of Thyroid Disease in Adults. Ann Surg [Internet]. 2020;271:399–410. Available from: https://journals.lww.com/10.1097/SLA.0000000000003735

19. AORN. Guidelines for perioperative practices. 2022 editi. Denver: Association of periOperative Registered Nurses; 2022. 1–1274 p.

20. Ling ML, Apisarnthanarak A, Abbas A, Morikane K, Lee KY, Warrier A, et al. APSIC guidelines for the prevention of surgical site infections. Antimicrob Resist Infect Control [Internet]. 2019;8:174. Available from: https://aricjournal.biomedcentral.com/articles/10.1186/s13756-019-0638-8

21. American Society of Anesthesiologists Task Force on Acute Pain Management. Practice Guidelines for Acute Pain Management in the Perioperative Setting. Anesthesiology [Internet]. 2012;116:248–73. Available from: https://pubs.asahq.org/anesthesiology/article/116/2/248/12956/Practice-Guidelines-for-Acute-Pain-Management-in

22. American Society of Anesthesiologists Task Force on Central Venous Access. Practice Guidelines for Central Venous Access 2020. Anesthesiology [Internet]. 2020;132:8–43. Available from: https://pubs.asahq.org/anesthesiology/article/132/1/8/108838/Practice-Guidelines-for-Central-Venous-Access

23. American Society of Anesthesiologists Task Force on Neuraxial Opioids and the American Society of Regional Anesthesia and Pain Medicine. Practice guidelines for the prevention, detection, and management of respiratory depression associated with neuraxial opioid. Vol. 124, Anesthesiology. Lippincott Williams and Wilkins; 2016. p. 535–52.

24. Apfelbaum JL, Hagberg CA, Connis RT, Abdelmalak BB, Agarkar M, Dutton RP, et al. 2022 American Society of Anesthesiologists Practice Guidelines for Management of the Difficult Airway. Anesthesiology [Internet]. 2022;136:31–81. Available from: https://pubs.asahq.org/anesthesiology/article/136/1/31/117915/2022-American-Society-of-Anesthesiologists

25. ASA. Practice Guidelines for Preoperative Fasting and the Use of Pharmacologic Agents to Reduce the Risk of Pulmonary Aspiration: Application to Healthy Patients Undergoing Elective Procedures. Anesthesiology [Internet]. 2017;126:376–93. Available from: https://pubs.asahq.org/anesthesiology/article/126/3/376/19733/Practice-Guidelines-for-Preoperative-Fasting-and

26. Bratzler DW, Dellinger EP, Olsen KM, Perl TM, Auwaerter PG, Bolon MK, et al. Clinical practice guidelines for antimicrobial prophylaxis in surgery. American Journal of Health-System Pharmacy [Internet]. 2013 [cited 2022 Sep 22];70:195–283. Available from: https://academic.oup.com/ajhp/article/70/3/195/5112717

27. Horlocker TT, Vandermeuelen E, Kopp SL, Gogarten W, Leffert LR, Benzon HT. Regional Anesthesia in the Patient Receiving Antithrombotic or Thrombolytic Therapy. Reg Anesth Pain Med [Internet]. 2018;43:263–309. Available from: https://rapm.bmj.com/lookup/doi/10.1097/AAP.0000000000000763

28. Benavides Caro CA, Prieto Alvarado FE, Torres M, Buitrago G, Gaitán Duarte H, García C, et al. Evidence-based clinical practice manual: Postoperative controls. Colombian Journal of Anesthesiology [Internet]. 2015;43:20–31. Available from: http://linkinghub.elsevier.com/retrieve/pii/S2256208714001266

29. Clark SC, Dunning J, Alfieri OR, Elia S, Hamilton LR, Kappetein AP, et al. EACTS guidelines for the use of patient safety checklists. European Journal of Cardio-Thoracic Surgery [Internet]. 2012;41:993–1004. Available from: https://academic.oup.com/ejcts/article-lookup/doi/10.1093/ejcts/ezs009

30. Frank U, Nikol S, Belch J, Boc V, Brodmann M, Carpentier PH, et al. ESVM Guideline on peripheral arterial disease. Vasa [Internet]. 2019;48:1–79. Available from: https://econtent.hogrefe.com/doi/10.1024/0301-1526/a000834

31. Halvorsen S, Mehilli J, Cassese S, Hall TS, Abdelhamid M, Barbato E, et al. 2022 ESC Guidelines on cardiovascular assessment and management of patients undergoing non-cardiac surgery. Eur Heart J [Internet]. 2022;43:3826–924. Available from: https://academic.oup.com/eurheartj/article/43/39/3826/6675076

32. Neumann FJ, Sousa-Uva M, Ahlsson A, Alfonso F, Banning AP, Benedetto U, et al. 2018 ESC/EACTS Guidelines on myocardial revascularization. Vol. 40, European Heart Journal. Oxford University Press; 2019. p. 87–165.

33. Pagano D, Milojevic M, Meesters MI, Benedetto U, Bolliger D, von Heymann C, et al. 2017 EACTS/EACTA Guidelines on patient blood management for adult cardiac surgery. European Journal of Cardio-Thoracic Surgery [Internet]. 2018 [cited 2022 Sep 22];53:79–111. Available from: http://academic.oup.com/ejcts/article/53/1/79/4316171

34. Sousa-Uva M, Head SJ, Milojevic M, Collet JP, Landoni G, Castella M, et al. 2017 EACTS Guidelines on perioperative medication in adult cardiac surgery. European Journal of Cardio-Thoracic Surgery [Internet]. 2018 [cited 2022 Sep 22];53:5–33. Available from: http://academic.oup.com/ejcts/article/53/1/5/4360955

35. Valgimigli M, Bueno H, Byrne RA, Collet JP, Costa F, Jeppsson A, et al. 2017 ESC focused update on dual antiplatelet therapy in coronary artery disease developed in collaboration with EACTS. Eur Heart J [Internet]. 2018;39:213–60. Available from: https://academic.oup.com/eurheartj/article/39/3/213/4095043

36. Wahba A, Milojevic M, Boer C, De Somer FMJJ, Gudbjartsson T, van den Goor J, et al. 2019 EACTS/EACTA/EBCP guidelines on cardiopulmonary bypass in adult cardiac surgery. European Journal of Cardio-Thoracic Surgery [Internet]. 2020;57:210–51. Available from: https://academic.oup.com/ejcts/advance-article/doi/10.1093/ejcts/ezz267/5579823

37. Royal College of Physicians Ireland. Prevention and Control Methicillin-Resistant Staphylococcus aureus (MRSA) [Internet]. 2013 [cited 2023 Feb 16]. Available from: https://assets.gov.ie/11637/16cc6fd28fb147bf8066dd2de902072e.pdf

38. Lepelletier D, Saliou P, Lefebvre A, Lucet JC, Grandbastien B, Bruyère F, et al. “Preoperative risk management: Strategy for Staphylococcus aureus preoperative decolonization” (2013 update). Med Mal Infect [Internet]. 2014;44:261–7. Available from: https://linkinghub.elsevier.com/retrieve/pii/S0399077X14000961

39. Malcolm J, Halperin I, Miller DB, Moore S, Nerenberg KA, Woo V, et al. In-Hospital Management of Diabetes. Can J Diabetes [Internet]. 2018 [cited 2023 Jul 18];42:S115–23. Available from: https://www.canadianjournalofdiabetes.com/issue/S1499-2671(17)X0005-1

40. Agency for Clinical Innovation (N.S.W.), Gastroenterological Nurses College of Australia. A clinician’s guide : caring for people with gastrostomy tubes and devices : from pre-insertion to ongoing care and removal. 2015. 89 p.

41. Ruiz Iban MA, Tejedor A, Gil Garay E, Revenga C, Hermosa JC, Montfort J, et al. Consenso GEDOS-SECOT sobre el proceso de atención a pacientes con artrosis de rodilla e indicación de artroplastia. Rev Esp Cir Ortop Traumatol [Internet]. 2017;61:296–312. Available from: https://linkinghub.elsevier.com/retrieve/pii/S1888441517300620

42. Ruiz Ibán MA, Maculé F, Torner P, Gil Garay E, Oteo-Álvaro A, López Millán JM, et al. SECOT-GEDOS consensus on pre-surgical pain management in knee and hip arthrosis. Rev Esp Cir Ortop Traumatol [Internet]. 2015;59:186–99. Available from: http://www.ncbi.nlm.nih.gov/pubmed/25435293

43. EAU. EAU Guidelines on Urological Infections [Internet]. Bonkat G, Bartoletti R, Bruyère F, Cai T, Geerlings SE, Köves B, et al., editors. Arnhem: European Association of Urology; 2022 [cited 2022 Sep 22]. Available from: https://uroweb.org/guidelines

44. EAU. EAU Guidelines on Chronic Pelvic Pain [Internet]. 2022 [cited 2023 Feb 15]. Available from: https://d56bochluxqnz.cloudfront.net/documents/full-guideline/EAU-Guidelines-on-Chronic-Pelvic-Pain-2022_2022-03-29-084111_kpbq.pdf

45. Matoses-Chirivella C, Navarro-Ruíz A, Lumbreras B. Development and validation of a guide for the continuity of care in perioperative medication management. Journal of Orthopaedics and Traumatology [Internet]. 2018;19:4. Available from: https://jorthoptraumatol.springeropen.com/articles/10.1186/s10195-018-0490-2

46. Anne S, Mims J “Whit,” Tunkel DE, Rosenfeld RM, Boisoneau DS, Brenner MJ, et al. Clinical Practice Guideline: Opioid Prescribing for Analgesia After Common Otolaryngology Operations. Otolaryngology–Head and Neck Surgery [Internet]. 2021;164:S1–42. Available from: https://onlinelibrary.wiley.com/doi/10.1177/0194599821996297

47. RCOA. GPAS. Chapter 13: Guidelines for the Provision of Ophthalmic Anaesthesia Services [Internet]. London; 2022. Available from: https://www.rcoa.ac.uk/gpas/chapter-13

48. RCOA. GPAS. Chapter 5: Guidelines for the Provision of Emergency Anaesthesia Services [Internet]. London; 2022. Available from: https://www.rcoa.ac.uk/gpas/chapter-5#glossary

49. RCOA. GPAS. Chapter 15: Guidelines for the Provision of Anaesthesia Services for Vascular Procedures [Internet]. London; 2022 [cited 2022 Dec 28]. Available from: https://www.rcoa.ac.uk/gpas/chapter-15

50. RCOA. GPAS. Chapter 14: Guidelines for the Provision of Neuroanaesthetic Services [Internet]. London; 2021 [cited 2022 Dec 28]. Available from: https://www.rcoa.ac.uk/gpas/chapter-14

51. RCOA. GPAS. Chapter 17: Guidelines for the Provision of Anaesthesia Services for Burn and Plastics Surgery [Internet]. London; 2022. Available from: https://www.rcoa.ac.uk/gpas/chapter-17

52. RCOA. GPAS. Chapter 6: Guidelines for the Provision of Anaesthesia Services for Day Surgery [Internet]. London; 2021 [cited 2023 Jun 6]. Available from: https://www.rcoa.ac.uk/node/18556

53. RCOA. GPAS. Chapter 12: Guidelines for the Provision of Anaesthesia Services for ENT, Oral Maxillofacial and Dental surgery [Internet]. London; 2022 [cited 2023 Feb 16]. Available from: https://www.rcoa.ac.uk/gpas/chapter-12

54. RCOA. GPAS. Chapter 11: Guidelines for the Provision of Anaesthesia Services for Inpatient Pain Management [Internet]. London; 2022 [cited 2023 Jun 6]. Available from: https://www.rcoa.ac.uk/gpas/chapter-11

55. RCOA. GPAS. Chapter 18: Guidance on the Provision of Anaesthesia Services for Cardiac and Thoracic Procedures [Internet]. London; 2021 [cited 2022 Dec 28]. Available from: https://www.rcoa.ac.uk/gpas/chapter-18#glossary

56. RCOA. GPAS. Chapter 16: Guidelines for the Provision of Anaesthesia Services for Trauma and Orthopaedic Surgery [Internet]. London ; 2021 [cited 2022 Sep 21]. Available from: https://www.rcoa.ac.uk/gpas/chapter-16

57. RCOA. GPAS. Chapter 2: Guidelines for the Provision of Anaesthesia Services for Perioperative Care of Elective and Urgent Care Patients [Internet]. London; 2022 [cited 2022 Sep 21]. Available from: https://www.rcoa.ac.uk/gpas/chapter-2

58. RCOA. GPAS. Chapter 9: Guidelines for the Provision of Anaesthesia Services for an Obstetric Population [Internet]. London; 2022 [cited 2023 Jun 6]. Available from: https://www.rcoa.ac.uk/gpas/chapter-9

59. Rubino F, Nathan DM, Eckel RH, Schauer PR, Alberti KGMM, Zimmet PZ, et al. Metabolic Surgery in the Treatment Algorithm for Type 2 Diabetes: A Joint Statement by International Diabetes Organizations. Surgery for Obesity and Related Diseases [Internet]. 2016;12:1144–62. Available from: https://linkinghub.elsevier.com/retrieve/pii/S1550728916301356

60. Secretaría de Salud. Intervenciones de Enfermería para la Atención del Adulto con Colelitiasis y Colecistitis Crónica Agudizada [Internet]. Centro Nacional de Excelencia Tecnológica en Salud, editor. Mexico DF: Secretaría de Salud; 2014. Available from: http://www.imss.gob.mx/profesionales/guiasclinicas/Pages/guias.aspx

61. Secretaría de Salud. Intervenciones de enfermería para la atención de la mujer con herida quirúrgica infectada post cesárea en los tres niveles de atención [Internet]. Mexico DF: Centro Nacional de Excelencia Tecnológica en Salud (CENETEC); 2018 [cited 2022 Sep 21]. Available from: http://www.cenetec-difusion.com/CMGPC/GPC-SS-824-18/ER.pdf

62. Secretaría de Salud. Diagnóstico y tratamiento de las Infecciones Asociadas a Dispositivos Ortopédicos Prótesis y/o material de osteosíntesis [Internet]. Centro Nacional de Excelencia Tecnológica en Salud (CENETEC), editor. México DF: Secretaría de Salud; 2013 [cited 2022 Sep 21]. Available from: http://www.imss.gob.mx/

63. IMSS. Prevención y Manejo de las Complicaciones Postoperatorias en Cirugía no Cardiaca en el Adulto Mayor [Internet]. Coordinación de Unidades Médicas de Alta Especialidad, editor. Mexico: Instituto Mexicano del Seguro Social; 2013 [cited 2022 Sep 21]. Available from: http://www.imss.gob.mx/profesionales/guiasclinicas/Pages/guias.aspx

64. IMSS. Intervenciones Preventivas Para la Seguridad en el Paciente Quirúrgico [Internet]. Coordinación de Unidades Médicas de Alta Especialidad, editor. Mexico: Instituto Mexicano del Seguro Social; 2013 [cited 2022 Sep 21]. Available from: http://www.imss.gob.mx/profesionales/guiasclinicas/Pages/guias.aspx

65. IMSS. Prevención, Diagnóstico y Tratamiento de dehiscencia completa de herida quirúrgica de abdomen en los tres niveles de atención [Internet]. Coordinación de Unidades Médicas de Alta Especialidad, editor. Mexico DF: Instituto Mexicano del Seguro Social; 2016 [cited 2022 Sep 21]. Available from: http://www.imss.gob.mx/

66. IMSS. Prevención y diagnóstico de la infección del sitio quirúrgico. Guía de Evidencias y Recomendaciones: Guía de Práctica Clínica [Internet]. Coordinación de Unidades Médicas de Alta Especialidad, editor. Mexico: Instituto Mexicano del Seguro Social; 2018 [cited 2022 Sep 21]. Available from: http://www.imss.gob.mx/

67. Lanas A, Benito P, Alonso J, Hernández-Cruz B, Barón-Esquivias G, Perez-Aísa A, et al. [Safe prescription recommendations for non steroidal anti-inflammatory drugs: Consensus document ellaborated by nominated experts of three scientific associations (SER-SEC-AEG)]. Gastroenterol Hepatol [Internet]. 2014;37:107–27. Available from: http://www.ncbi.nlm.nih.gov/pubmed/24529572

68. Bischoff SC, Austin P, Boeykens K, Chourdakis M, Cuerda C, Jonkers-Schuitema C, et al. ESPEN guideline on home enteral nutrition. Clinical Nutrition [Internet]. 2020;39:5–22. Available from: https://linkinghub.elsevier.com/retrieve/pii/S0261561419301980

69. Department of Health. Nutrition screening and use of oral nutrition support for adults in the acute care setting [Internet]. Dublin; 2020 [cited 2022 Dec 28]. (22). Report No.: 22. Available from: http://health.gov.ie/en/collection/c9fa9a-national-clinical-guidelines/

70. Cagle S, Hutcherson B, Wiley A. Health Care-Associated Infections: Best Practices for Prevention. 2022 [cited 2023 Feb 16]; Available from: https://pubmed.ncbi.nlm.nih.gov/35289570/

71. Horn EP, Klar E, Höcker J, Bräuer A, Bein B, Wulf H, et al. [Prevention of perioperative hypothermia : Implementation of the S3 guideline]. Der Chirurg [Internet]. 2017;88:422–8. Available from: http://link.springer.com/10.1007/s00104-016-0357-0

72. RNAO. Clinical Best Practice Guidelines Care Transitions [Internet]. 2014. Available from: www.rnao.ca/bestpractices

73. Busetto L, Dicker D, Azran C, Batterham RL, Farpour-Lambert N, Fried M, et al. Practical Recommendations of the Obesity Management Task Force of the European Association for the Study of Obesity for the Post-Bariatric Surgery Medical Management. Obes Facts [Internet]. 2017;10:597–632. Available from: https://www.karger.com/Article/FullText/481825

74. ACOG. ACOG PRACTICE BULLETIN Clinical Management Guidelines for Obstetrician-Gynecologists Prevention of Infection After Gynecologic Procedures [Internet]. 2018 [cited 2023 Feb 16]. Available from: https://pubmed.ncbi.nlm.nih.gov/29794678/
